# Identification of UDP-dependent glycosyltransferases in the wallflower cardenolide biosynthesis pathway

**DOI:** 10.1016/j.jbc.2025.108565

**Published:** 2025-04-30

**Authors:** Owen S. Patrick, Gordon C. Younkin, Rebecca G. Brody, Jessica W. Hem, Georg Jander, Cynthia K. Holland

**Affiliations:** 1 Department of Biology, Williams College, Williamstown, Massachusetts, USA; 2 Plant Biology Section, School of Integrative Plant Science, Cornell University, Ithaca, New York, USA; 3 Boyce Thompson Institute, Ithaca, New York, USA

**Keywords:** glycosyltransferase, plant biochemistry, secondary metabolism, enzyme kinetics, mass spectrometry

## Abstract

Cardenolides are potent plant-defensive metabolites that have been studied for decades for their significance in plant–insect interactions and their use in treating heart failure in humans. With recent advancements in genome and transcriptome sequencing, genes in the cardenolide biosynthetic pathway have begun to be identified. Here we employed gene co-expression network analysis using published data from the cardenolide-producing plant *Erysimum cheiranthoides* (wormseed wallflower) to identify two UDP-dependent glycosyltransferases, UGT73C44 and UGT73C45, that are capable of glucosylating the aglycone cardenolide digitoxigenin as well as other predicted cardenolide pathway intermediates. *In vitro* and *in planta* assays revealed that UGT73C44 acted on cardenolide pathway intermediates with a low *K*_m_ value of 7.0 μM for digitoxigenin, while UGT73C45 displayed broader substrate specificity *in vitro* and could glucosylate diverse steroid and flavonoid substrates. Phylogeny and comparisons of structural models of UGT73C44 and UGT73C45 suggest that the enzymes have divergent active site architectures, which may account for their different substrate specificities. These data report the first plant-derived UGT specific to cardenolides, advancing our understanding of cardenolide biosynthesis and the enzymes that drive specialized metabolite diversity. These findings lay the foundation for future efforts to reconstitute the cardenolide pathway in heterologous systems and design cardenolide analogs with the potential for improved therapeutic properties.

Cardenolides are plant-defensive metabolites that have evolved independently in several plant families, including in the genus *Erysimum* (wallflowers; Brassicaceae) (1, 2). Cardenolides act as potent allosteric inhibitors of the Na^+^/K^+^-ATPase, which imparts their toxicity to herbivores (3). However, several orders of insects have evolved resistance-conferring mutations in their Na^+^/K^+^-ATPase, allowing them to tolerate and sequester cardenolides (4). In mammals, low doses of the cardenolide digoxin have been used to increase the contractile force of the heart in patients with chronic heart failure (5). In addition to treating heart disease, cardenolides have demonstrated apoptotic and senolytic activity in cancer cells, suggesting that they may be useful antitumor therapeutics (6, 7). While digoxin is on the World Health Organization's Model List of Essential Medications, the medication is still sourced from plants, and the majority of the genes responsible for cardenolide biosynthesis in plants remain to be identified (8).

Structurally, cardenolides consist of a 6-6-6-5-membered steroid core with an unsaturated 5-membered lactone ring and 0 to 4 sugars added to the hydroxyl on C-3 (Fig. 1). The simplest aglycone cardenolide in plants is digitoxigenin, which has hydroxyl groups on C-3 and C-14, and digitoxigenin is a precursor to additional 5β-cardenolides, which include the pharmaceutical digoxin (9). *Erysimum cheiranthoides* (wormseed wallflower) has been shown to produce at least 15 unique aglycones, and at least 10 unique monosaccharides are incorporated into cardenolide glycosides, including sugars found in a variety of plants, such as glucose, rhamnose, and xylose, and specialized sugars found exclusively in cardenolide-producing species, such as digitoxose (10). The bioactivity of these diverse cardenolides and their contributions to plant defense against herbivores remain to be investigated.

**Figure 1:**
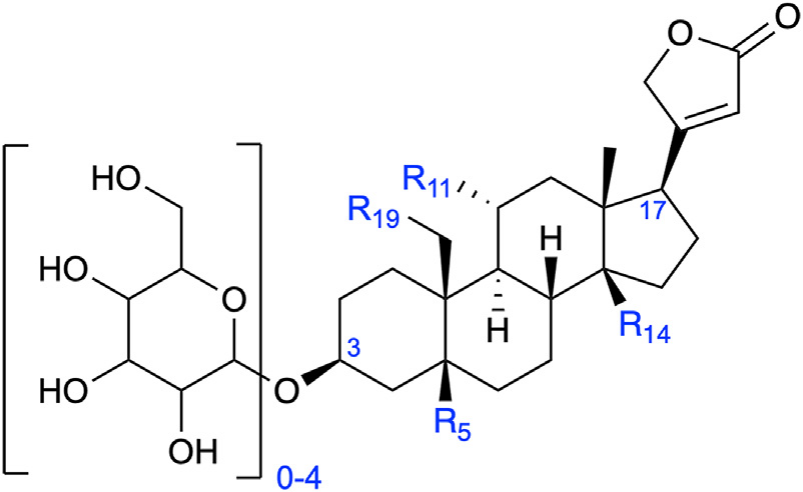
**Cardenolide structure in *E. cheiranthoides*.** Cardenolides are 6-6-6-5-membered rings with 0 to 4 sugars added to C-3 and a 5-membered lactone ring at C-17. Sites where functional groups (*i.e.*, hydroxyl groups or aldehydes) are located are labeled as R.

Recent work has begun to identify enzymes responsible for the early steps in cardenolide biosynthesis upstream of digitoxigenin from *Digitalis* spp. (foxglove) and *E. cheiranthoides* (9, 11) (Fig. 2*A*). In these cardenolide-producing plants, the cytochrome P450 monooxygenase CYP87A acts as a side-chain cleavage enzyme to generate the cardenolide precursor pregnenolone. Next, expression of this P450 (EcCYP87A126, or CARD1) with three additional wallflower enzymes [Ec3βHSD (CARD2), Ec3KSI (CARD3), and EcP5βR2 (CARD4)] in *Nicotiana benthamiana* has been shown to generate epipregnanolone (12). Successive hydroxylation by two 2-oxoglutarate-dependent dioxygenases forms 14β,21-dihydroxyepipregnanolone, the precursor to 5β-cardenolides in wallflowers (13). The enzymes responsible for lactone ring formation have not yet been identified.

**Figure 2:**
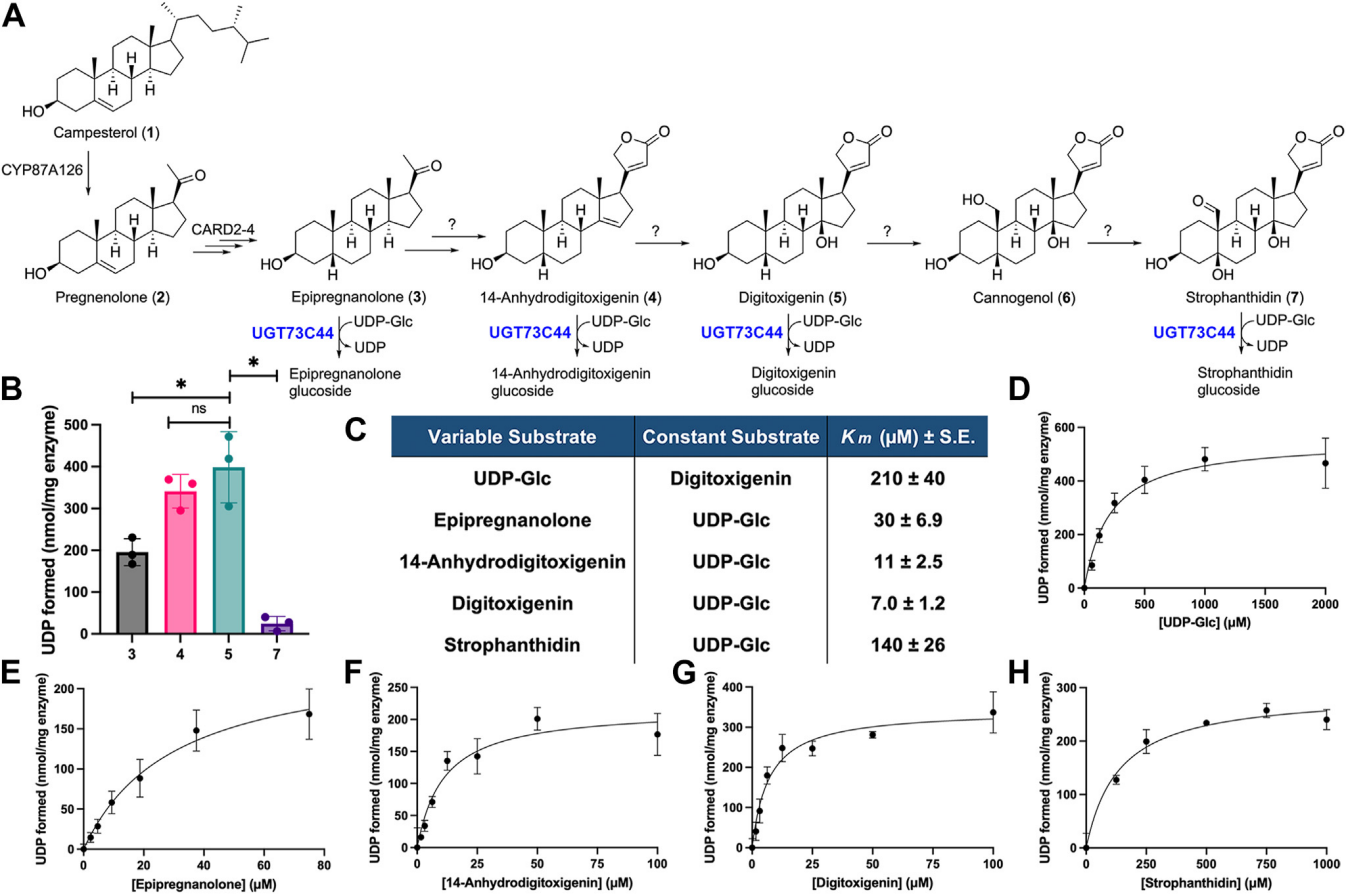
**UGT73C44 glycosylates multiple cardenolide pathway intermediates.***A*, UGT73C44 catalyzes the glycosylation of **3**, **4**, **5**, and **7** using UDP-Glc as a sugar donor. *B*, activity of UGT73C44 with **3**, **4**, **5**, and **7** was determined by reactions with 350 ng of enzyme, 0.1 mM cardenolide substrate, and 1 mM UDP-Glc incubated for 10 min. The asterisk denotes a *p*-value < 0.05, while ns denotes no significance (α level of 0.05). *p*-values were determined using an unpaired, two-tailed *t* test with Welch's correction in GraphPad Prism 10. *C*, Michaelis-Menten constants (*K*_m_) were determined by varying the concentration of one substrate (variable substrate) while holding the concentration of the other substrate constant (constant substrate) and fitting activity data to the Michaelis-Menten model. *D*, Michaelis-Menten plot of UGT73C44 activity with 0 to 2 mM UDP-Glc determined using 350 ng of enzyme and 0.4 mM **5** incubated for 10 min. Michaelis-Menten plots of UGT73C44 activity with: *E,* 0 to 75 μM **3** determined using 1250 ng of enzyme and incubated for 12 min; *F,* 0 to 0.1 mM **4** determined using 350 ng of enzyme and incubated for 10 min; *G*, 0 to 0.1 mM **5** determined using 350 ng of enzyme and incubated for 10 min; and *H*, 0 to 1 mM **7** determined using 1250 ng of enzyme and incubated for 12 min. All reactions were conducted at 37 °C, and data are expressed as mean ± s.d. (n = 3).

In wallflower, additional hydroxylations at the carbons at positions 5, 11, and 19, along with the hydroxyl group at C-14 on digitoxigenin, give rise to other cardenolide genins, including cannogenol, which is hydroxylated at C-19, and strophanthidin, which has a hydroxyl group at C-5 and an aldehyde at C-19 (10) (Fig. 1). The hydroxyl group at C-19 is added by a cytochrome P450 monooxygenase (14). A key feature of the cardenolides, or cardiac glycosides, is the addition of one or more sugars to C-3, as glycosylation increases the binding affinity of cardenolides for the Na^+^/K^+^-ATPase, and the addition of sugars increases isoform selectivity for the α2 subunit, which is expressed in cardiac myocytes and plays a role in muscle contractility and blood pressure regulation (15, 16). Glycosylation is likely carried out by UDP-dependent glycosyltransferases (UGTs), which catalyze the addition of UDP-activated sugars onto substrates, thus contributing to the structural diversity of cardenolides (17, 18).

As glycosylation is a key step in forming bioactive cardenolides, we set out to identify UGTs that would glycosylate digitoxigenin. It has been observed that *E. cheiranthoides* produces at least 28 distinct monoglycosylated and diglycosylated digitoxigenin, cannogenol, and strophanthidin structures, but additional glycosylated cardenolides may also be present (10). The cytochrome P450s and UGTs that catalyze the modification of the cardenolide core have not been identified in any cardenolide-producing species, and the order of these reactions has yet to be determined. The cardenolide biosynthetic pathway has escaped investigation for decades due to a lack of genomic resources. However, a recent high-quality genome and transcriptome from *E. cheiranthoides* has aided in candidate gene identification (10). Using gene co-expression analysis, here we identified two genes in the UGT73C family, *Erche07g009482* (UGT73C44) and *Erche07g009500* (UGT73C45), that can glycosylate digitoxigenin and strophanthidin (12). While UGT73C45 has broad substrate specificity and glycosylates a wide range of steroid and flavonoid substrates, UGT73C44 has vanishingly low activity with the flavonoids tested and thus appears to be specific for cardenolide pathway intermediates.

## Results

### Identification of candidate UGTs in *E. cheiranthoides*

**Figure 3:**
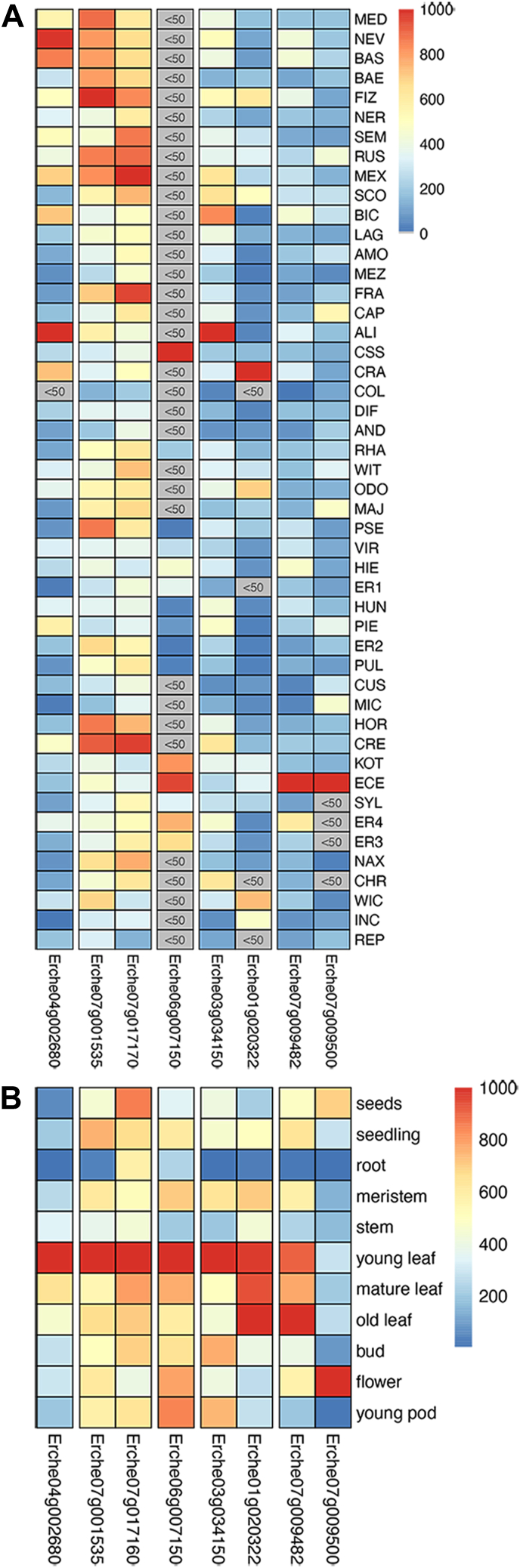
**Gene expression profile across *Erysimum* species and tissues.** Expression of six known cardenolide biosynthesis genes (*CARD1*: *Erche04g002680; CARD2: Erche07g001535; CARD3: Erche07g017160; CARD4: Erche06g007150; CARD5: Erche03g034150; CARD6: Erche01g020322*) and two candidate UGTs (*Erche07g009482* and *Erche07g009500*) across *(A)* 48 *Erysimum* species and *(B) Erysimum cheiranthoides* tissues. *Gray* indicates very low expression in species dataset (<50 counts per million reads). In both datasets, expression for each gene is normalized to a maximum value of 1000 to allow for visual comparisons across genes. N = 1 replicate per species, and n = 3 to 4 replicates per tissue.

In searching for genes upstream of digitoxigenin, we previously identified a cardenolide-related biosynthetic gene co-expression cluster from an analysis of 48 *Erysimum* species (12, 19). Four of the genes in the cluster were confirmed to contribute to digitoxigenin biosynthesis. This cluster also contained a glycosyltransferase, *Ec07g009482.1*, which we hypothesized may also contribute to cardenolide biosynthesis. We noticed that a neighboring gene 14 kbp away in the *E. cheiranthoides* genome, *Ec07g009500.1*, was also predicted to be a glycosyltransferase (10). Both genes were expressed across the 48 *Erysimum* species and were expressed at high levels in *E. cheiranthoides* (Fig. 3*A*). They are also widely expressed across *E. cheiranthoides* tissues, with the highest expression in tissues where cardenolides accumulate: leaves in the case of *Ec07g009482.1*, and flowers in the case of *Ec07g009500.1* (Fig. 3*B*). Therefore, we reasoned that these two genes were strong candidates for cardenolide pathway glycosyltransferases and proceeded to characterize them *in vitro*. Genes were submitted for naming by the UGT naming committee, and the glycosyltransferase encoded by *Ec07g009482.1* was named UGT73C44, and the protein encoded by *Ec07g009500.1* was named UGT73C45 (Fig. S1).

Based on phylogeny, UGT73C44 is most closely related to the *Arabidopsis thaliana* UGT73C1 and UGT73C7 (Fig. S2). Six of the seven *Arabidopsis* UGT73Cs are clustered on chromosome 2, and several have been functionally characterized. EcUGT73C44 shares an ancestor with AtUGT73C1, which has *O-*glucosyltransferase activity with several cytokinins, including dihydrozeatin-9-*N*-glucoside, dihydrozeatin, and *cis*- and *trans*-zeatin (20). The closely related *AtUGT73C7* gene is upregulated in response to *Pseudomonas syringae pv. tomato* infection, and the encoded protein glucosylates two hydroxycinnamic acids upstream of phenylpropanoid metabolism, *p*-coumaric acid and ferulic acid (21). EcUGT73C45 is more closely related to the *Arabidopsis* UGT73C5 and UGT73C6. Both AtUGT73C5 and AtUGT73C6 glucosylate the brassinosteroid hormone brassinolide and its precursor, castasterone, forming 23-*O*-glucosides (22, 23). While the *Arabidopsis* UGT73C2 remains to be functionally characterized, UGT73C3 and UGT73C4 are also induced by *P. syringae* infection and glucosylate the lignan pinoresinol to form pinoresinol monoglucoside and diglucoside with weak activity toward the flavonoids quercetin, apigenin, and naringenin (24). Given the wide range of substrates accepted by UGT73C enzymes, simple phylogenetic comparisons were insufficient to infer the functions of the two *Erysimum* UGTs.

### UGT73C44 and UGT73C45 glucosylate cardenolide pathway intermediates *in vitro*

To test candidate UGTs for activity with aglycone cardenolides, we heterologously expressed *UGT73C44* and *UGT73C45* in *Escherichia coli,* and the resulting proteins were initially screened for glucosylation of four commercially available compounds that are putative pathway intermediates: epipregnanolone **(3)**, 14-anhydrodigitoxigenin **(4)**, digitoxigenin **(5)**, and strophanthidin **(7)** (Fig. 2, *A* and *B*). Using an endpoint luminescence-coupled assay, nmol of UDP product formed were determined per mg of enzyme. We detected UDP formation with all four steroids to varying degrees with UGT73C44 when UDP-glucose (UDP-Glc) was saturating using 100 μM of **3, 4, 5,** or **7** (Fig. 2*B*). In comparing the mean product formation, UGT73C44 formed the highest amount of product with 5 as a substrate (398 nmol/mg). While product formation was 15% lower with **4** (341 nmol/mg), there was no statistically significant difference between the means (*p* = 0.37). Further upstream in the predicted biosynthetic pathway, UGT73C44 demonstrated activity with **3** (196 nmol/mg), but approximately half as much product was formed as with **5** (*p* = 0.04). UGT73C44 has the lowest levels of product formation with **7** (24.7 nmol/mg), representing a 94% decrease compared to **5** (*p* = 0.01).

Before comparing the kinetic parameters of UGT73C44 with these four substrates, we first held the concentration of **5** constant and varied UDP-Glc concentration to determine its Michaelis-Menten constant (Fig. 2, *C* and *D*). The *K*_m_ value was 210 μM, and product formation plateaued with UDP-Glc concentrations of 1000 μM and higher. Because this is an endpoint assay, we were unable to calculate the turnover number (*k_cat_*) for any of our kinetic experiments. When varying the concentrations of **3, 4, 5,** or **7**, the UDP-Glc concentration was held constant at 1 mM. UGT73C44 has a *K*_m_ value of 7.0 μM with **5**, which was the lowest *K*_m_ value of any tested substrate (Fig. 2*C*). This low *K*_m_ value, coupled with the high levels of product formation when substrates were compared side-by-side, suggests that this is the primary substrate of this enzyme. Low *K*_m_ values of 11 μM and 30 μM were observed with **4** and **3**, respectively, and the substrate with the lowest levels of product formation, **7**, also had the highest Michaelis-Menten constant (140 μM) (Fig. 2, *C,* *E*, *F*, and *H*). Taken together, these kinetics data suggest that UGT73C44 is a UDP-dependent glycosyltransferase that may function in cardenolide biosynthesis in *E. cheiranthoides*. This represents the first identification of a cardenolide-specific UGT from any plant.

**Figure 4:**
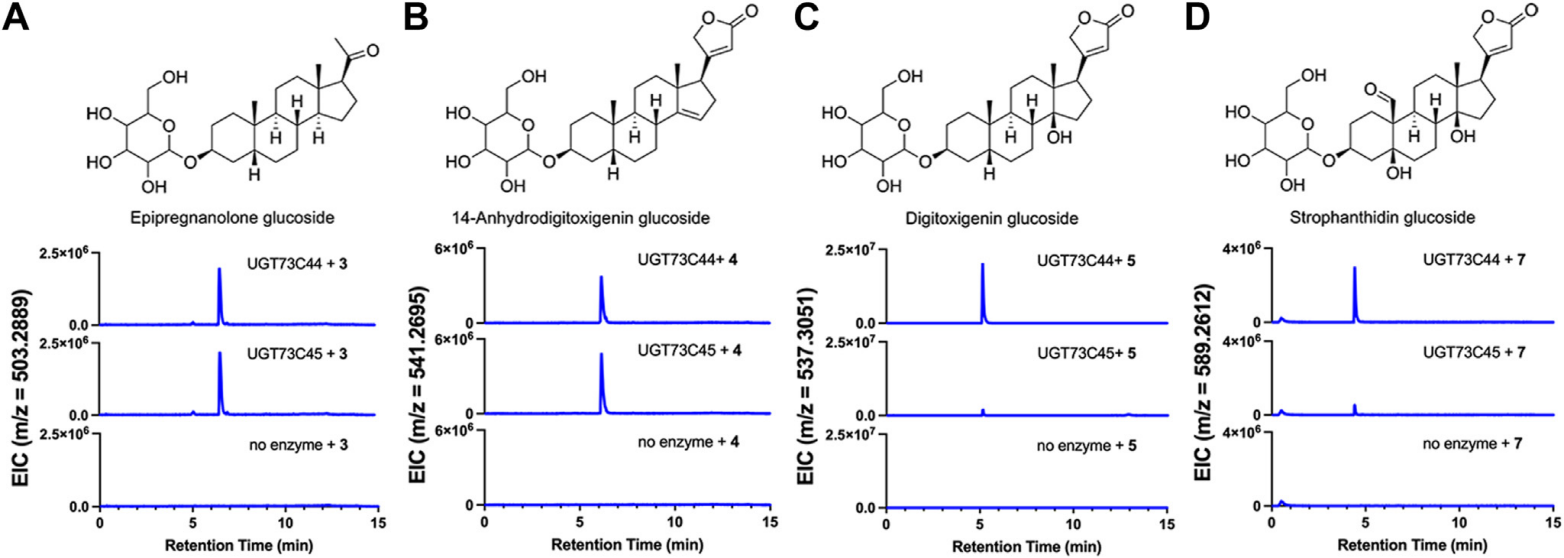
**Detection of glucosylated cardenolide pathway intermediates produced by UGT73C44 and UGT73C45 *in vitro*.***A,* extracted ion chromatogram (EIC) for an m/z that corresponds to glucosylated **3** [M+Na]^+^ is detected at a retention time of 6.55 min. *B*, EIC for an m/z that corresponds to glucosylated **4** [M+Na]^+^ is detected at a retention time of 6.15 min. *C*, EIC for an m/z that corresponds to glucosylated **5** [M+H]^+^ is detected at a retention time of 5.13 min. *D*, EIC for an m/z that corresponds to glucosylated **7** [M+Na]^+^ is detected at a retention time of 4.41 min. Data are representative of three technical replicates. Based on known cardenolide structures, we hypothesize that the steroid core is glucosylated at C-3, with predicted structures shown above the chromatograms.

When we assayed UGT73C45 in the same endpoint luminescence-coupled assay, the enzyme-only, no-substrate controls produced luminescence values that exceeded the standard curve, which meant that we were unable to reliably detect activity for this enzyme with any substrates. Given this, we reasoned that the sensitivity of LC-MS may permit us to detect product formation from an *in vitro* reaction. We also tested UGT73C44 in these mass spectrometry assays to confirm the formation of glucosylated products. Initially, we tested UGT73C44 and UGT73C45 with UDP-Glc and **3, 4, 5,** or **7**. These experiments confirmed that UGT73C44 glucosylates **3, 4, 5,** and **7**, and peaks were detectable at the same retention times with UGT73C45 for each of the four substrates (Figs. 4; S3 and S4). This confirmed that UGT73C45 is indeed active and can also glucosylate the same cardenolide pathway intermediates as UGT73C44 *in vitro*.

We also tested a steroid with 5α stereochemistry, (3β,5α)-3,21-dihydroxy-pregnan-20-one (3β,5α-THDOC), and peaks were detected for glucosylated products with both UGT73C44 and UGT73C45 (Fig. S5). In the extracted ion chromatograms for UGT73C45 with 3β,5α-THDOC, an extra peak appeared that was not in the chromatograms for the reactions with UGT73C44. Because there are two hydroxyl groups on 3β,5α-THDOC, it is possible that the enzyme may glucosylate both positions, but this was unable to be confirmed in the absence of standards.

### UGT73C45 glucosylates a diverse range of flavonoid and steroid substrates *in vitro*

Because UGTs in other plants have been found to have relatively broad specificity and glycosylate several classes of substrates, such as UGT71G1 from *Medicago truncatula* that accepts both steroids and flavonoids, we wondered whether these UGT73C enzymes from wallflower are also active with flavonoids (25). Furthermore, other UGT73Cs have demonstrated activity with flavonoids. Namely, the *A. thaliana* UGT73C6 is active with many aglycone and monoglycosylated flavonoids, including quercetin and quercetin-3-*O*-rhamnoside with UDP-glucose as a donor substrate (26). AtUGT73C6 is closely related to the EcUGT73C45, so we hypothesized that EcUGT73C45 may glucosylate quercetin and structurally similar flavonoids (Fig. S2).

**Figure 5:**
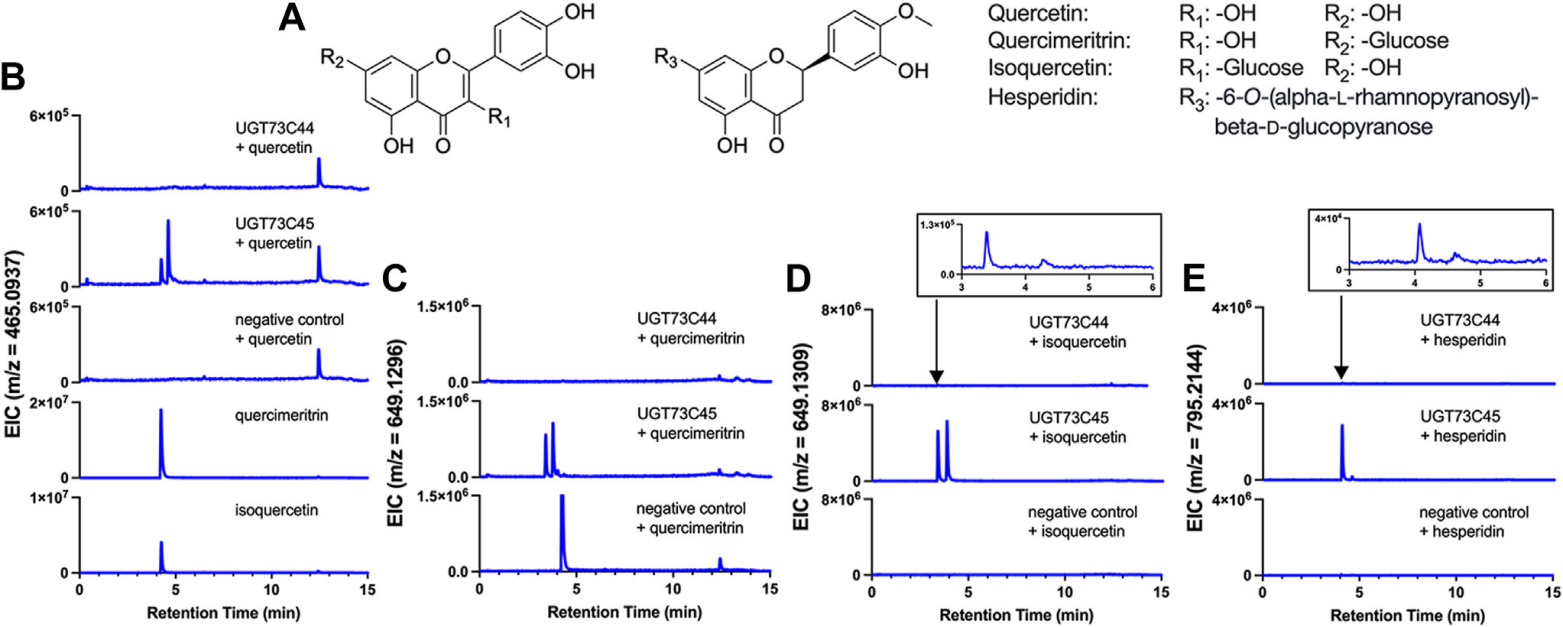
**Detection of glucosylated flavonoids produced by UGT73C44 and UGT73C45 *in vitro*.***A,* chemical structures of flavonoids used as substrates. Quercetin, quercimeritrin and isoquercetin use the core of the structure on the left, while hesperidin uses the structure on the right. *B*, extracted ion chromatogram (EIC) for an m/z that corresponds to glucosylated quercetin [M+H]^+^ is detected at retention times of 4.24 and 4.61 min. Standards for quercimeritrin and isoquercetin have retention times of 4.22 and 4.25 min, respectively. *C,* EIC for an m/z that corresponds to glucosylated quercimeritrin [M+Na]^+^ is detected at retention times of 3.39, 3.76, and 4.00 min. *D,* EIC for an m/z that corresponds to glucosylated isoquercetin [M+Na]^+^ is detected at retention times of 3.39 and 3.86 min. *E,* EIC for an m/z that corresponds to glucosylated hesperidin [M+Na]^+^ is detected at retention times of 4.07 and 4.59 min. Only minor product formation was detected for UGT73C44. Data are representative of three technical replicates.

To probe the substrate specificity of UGT73C44 and UGT73C45, we incubated the purified enzymes with potential flavonoid substrates and used LC-MS to detect product formation (Figs. 5; S6). Notably, UGT73C45 glucosylated flavonoids, including the pentahydroxyflavone quercetin (Fig. 5, *A* and *B*). In the extracted ion chromatograms for the m/z value that corresponds to the H^+^ adduct of quercetin glucoside, we noticed two peaks appear at 4.24 and 4.61 min, which we hypothesized may be the result of glucosylation at different positions. We included two standards, quercimeritrin (quercetin 7-*O*-β-D-glucoside) and isoquercetin (quercetin 3-*O*-β-D-glucopyranoside), which both had a retention time similar to the early-eluting peak at 4.24 min. To probe the substrate specificity further, we also tested both quercimeritrin and isoquercetin as substrates of UGT73C44 and UGT73C45 (Fig. 5, *C* and *D*). Again, two peaks were detected in the extracted ion chromatograms for the H^+^ adducts that are absent in the no-enzyme controls, suggesting that both monoglycosylated flavonoids can serve as substrates for UGT73C45. We also tested hesperidin, which is a trihydroxyflavone with a disaccharide on C-7, and for UGT73C45, a peak appeared that corresponds to the Na^+^ adduct of hesperidin glucoside (Fig. 5*E*). This suggests that UGT73C45 has broad substrate specificity and can accept both steroids and flavonoids, including monoglycosylated and diglycosylated flavonoids, as substrates (Figs. 4 and 5). However, UGT73C44 had vanishingly low activity with all flavonoids that were tested (Fig. S6). We were also unable to detect activity of UGT73C44 with flavonoids using the *in vitro* luminescence-coupled assay, further suggesting that this enzyme is largely unable to accept these flavonoids as substrates.

### *In planta* confirmation of UGT73C44 activity

**Figure 6:**
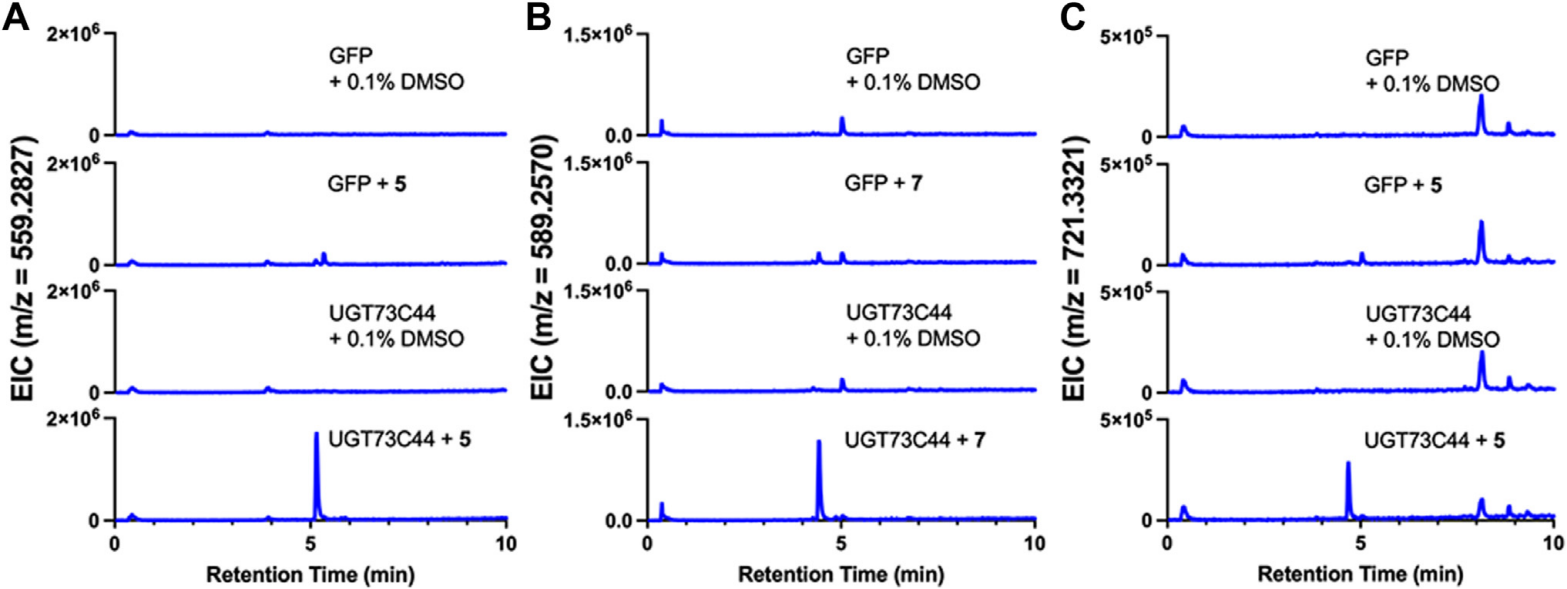
***In planta d*etection of glycosylated cardenolide pathway intermediates produced by UGT73C44 in *N. benthamiana*.***A,* extracted ion chromatogram (EIC) for an m/z that corresponds to glycosylated **5** [M+Na]^+^ is detected at a retention time of 5.15 min. *B*, EIC for an m/z that corresponds to glycosylated **6** [M+Na]^+^ is detected at a retention time of 4.42 min. *C*, EIC for an m/z that corresponds to diglycosylated **5** [M+Na]^+^ is detected at a retention time of 4.68 min. Data are representative of five technical replicates.

As UGT73C44 seems to be primarily involved in cardenolide biosynthesis *in vitro*, we sought to confirm its activity *in planta.* To do this, we cloned the wallflower *UGT73C44* gene into the pEAQ expression vector for *Agrobacterium*-mediated expression in *N. benthamiana* leaves. Four days post-infiltration, leaves were infiltrated with substrates—**3, 5, 7**, or a solvent control. On Day 5, leaves were harvested for product detection by LC-MS. The resulting data confirm that UGT73C44 is active *in planta* and can glucosylate digitoxigenin and strophanthidin (Figs. 6, *A* and *B*; S7). Given the broad substrate specificity of plant UGTs, it was unsurprising to detect peaks corresponding to monoglycosides of **5** and **7** in samples from leaves expressing *GFP* that had been infiltrated with either **5** or **7**, respectively, suggesting that *N. benthamiana* UGTs can glycosylate both **5** and **7**. However, the peaks were much more pronounced in leaves expressing *UGT73C44*. We did not see peaks corresponding to the glycosylation of **3** (Fig. S8). If there are other enzymes in *Nicotiana* that have a higher affinity for **3** that in some way modify the compound (*e.g.*, hydroxylation, glycosylation, *etc.*), then EcUGT73C44 might not be able to glycosylate a detectable amount of **3** before it is diverted to other pathways.

Out of curiosity, we searched the *in planta* data for mass features corresponding to diglycosides of both **5** and **7**, and a peak was detected in the extracted ion chromatograms that appeared only in the presence of **5** and UGT73C44 (Figs. 6*C*; S7). This peak was absent in chromatograms from the GFP leaves that were infiltrated with **5**.

When we searched the *in vitro* mass spectrometry data for peaks corresponding to diglucosides of **5**, small peaks were visible for both enzymes (Fig. S9). Peaks for diglucosylated **7** were only visible with UGT73C45. In the *in vitro* data, only small peaks were detected for the diglucosides. The retention time of the diglycoside of **5** *in planta* (4.68 min) differs from that detected *in vitro* (5.10 min), suggesting that the UDP-sugar donor *in planta* may be an isomeric hexose sugar other than glucose. Given that the *in vitro* reactions were incubated for 10 min in comparison to the 24-h length of the *in planta* reaction, the reaction times may contribute to this activity with the monoglycoside. However, there may be UGTs in *N. benthamiana* that can glycosylate the monoglycosides of **5** and **7**.

When we searched the *in vitro* mass spectrometry data for diglucosylated 3β,5α-THDOC, a substantial peak was detected for UGT73C45, but only a small peak was detected for UGT73C44 (Fig. S5). Together, these data suggested that UGT73C45 and UGT73C44, to a lesser extent, may also function in adding a second sugar onto monoglycosylated cardenolides.

To determine whether UGT73C44 and UGT73C45 could accept cardenolides containing one or two sugars as substrates, we repeated the *in vitro* LC-MS assay with both enzymes using ouabain, which has one sugar (L-rhamnose) on C-3, and periplocin, which has a diglycoside (D-cymarose and D-glucose) on C-3 (Fig. S10). Activity was not detected for either compound with either enzyme. However, future experiments may elucidate the chain-elongating activities of these UGTs and their significance in cardenolide production *in vivo*.

### Active site comparisons between UGT73C44 and UGT73C45

**Figure 7:**
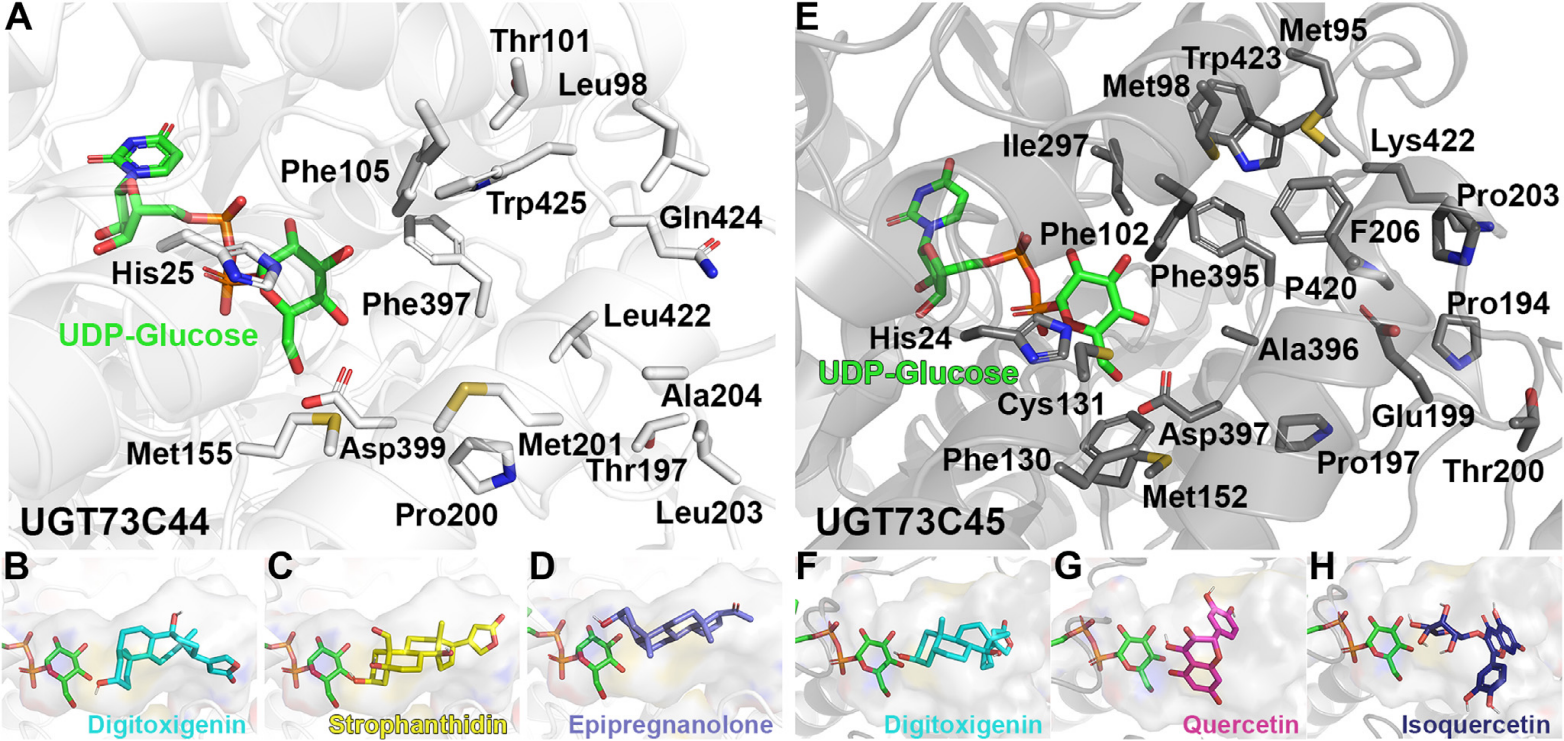
**Structural comparisons between UGT73C44 and UGT73C45.***A*, UDP-Glucose (*green*) was docked into the AlphaFold-generated structural model for UGT73C44 to identify putative active site residues. Docking was used to model how *B,* digitoxigenin **(5)**, *C*, strophanthidin **(7)**, and *D,* epipregnanolone **(3)** bind in the UGT73C44 active site pocket. *E*, UDP-Glucose (*green*) was docked into the AlphaFold-generated structural model for UGT73C45 to identify putative active site residues. Docking was used to model how *F,* digitoxigenin **(5)**, *G*, quercetin, and *H,* isoquercetin bind in the UGT73C45 active site pocket.

Although UGT73C44 and UGT73C45 are phylogenetically related, they exhibit key differences in substrate specificities. To understand the structural underpinnings of their activities, we used AlphaFold structural models of the proteins to visualize their predicted active sites with UDP-Glc bound (Fig. 7, *A* and *E*). Both proteins have a conserved catalytic dyad consisting of His and Asp (His25 and Asp399 in UGT73C44, and His24 and Asp397 in UGT73C45) (27). Both also have a number of hydrophobic residues that likely enable interactions with the less polar substrates, like **3** and **4**. The active site pocket of UGT73C44 has three aromatic residues (Phe105, Phe397, and Trp425), and the UGT73C45 active site has five aromatic residues (Phe102, Phe130, Phe206, Phe395, and Trp423), which may facilitate π-π stacking with conjugated π systems such as those found in flavonoids.

These UDP-Glc-bound structural models were used for molecular docking with relevant substrates, including digitoxigenin, strophanthidin, and epipregnanolone for UGT73C44 and digitoxigenin, quercetin, and isoquercetin for UGT73C45. While docking is using a static model and not an empirically solved structure, a sense of the active site architecture can be gleaned from comparisons between the two proteins. Notably, the size and shape of the cavities appear to vary, with UGT73C44 having a more cylindrical pocket and UGT73C45 having a more cavernous, globular active site (Fig. 7). These physical differences may allow UGT73C45 to accommodate larger and more structurally diverse substrates, including monoglycosylated steroids and flavonoids, and may constrain UGT73C44 to steroid substrates.

Aside from the catalytic dyad, there are no charged residues in the UGT73C44 active site, but there are three uncharged polar residues: Thr101, Thr197, and Gln424 (Fig. 7*A*). Situated closest to the solvent-exposed surface, Gln424 may facilitate hydrogen bond interactions between the hydroxyl on C-14 or the oxygens in the lactone ring of cardenolides or the ketone on epipregnanolone (Fig. 7, *A*–*D*). In the UGT73C45 active site, there are two charged residues, Glu199 and Lys422, in addition to the catalytic dyad (Fig. 7*E*). There is one additional polar residue, Thr200. These polar residues may contribute to interactions with polar flavonoids like quercetin and isoquercetin (Fig. 7, *G* and *H*).

A common structural feature in plant UGTs is the 44-amino acid plant secondary product GT (PSPG) motif, which includes residues that interact with the sugar donor and contribute to sugar specificity (28, 29). Others have found that a Gln at position 44 contributes to activity with UDP-Glc, and both UGT73C44 and UGT73C45 have a Gln at this position (30) (Fig. S11). An active site Arg proximal to the UDP-sugar donor has been shown to promote UDP-glucuronic acid activity, but an Arg is not found in either the UGT73C44 or UGT73C45 active site (31, 32). This sequence information is consistent with the activity that we see with both enzymes with UDP-Glc as a sugar donor.

## Discussion

While cardenolides have been of interest for centuries for treating ailments and for decades as a pharmaceutical, the genomic tools for pathway identification have only recently been made available for wallflower (*Erysimum* spp.), milkweed (*Asclepias* spp.), and foxglove (*D.* spp.) (9, 10, 11, 33). Having the resources to correlate tissue-level gene expression to metabolite abundance has enabled rapid advancements in elucidating the biosynthetic pathways of plant specialized metabolites (34, 35, 36). Here we have taken advantage of a wealth of resources available for *E. cheiranthoides* to characterize a UGT, UGT73C44, that generates cardenolides *in vitro* and *in planta*.

UGT73C44 had the highest activity and lowest Michaelis-Menten constant for **5**, followed by **4**, which lacks a hydroxyl on C-14, and **3**, which does not have the 5-membered lactone ring (Fig. 2, *A*–*C*). There was a marked decrease in activity and increase in the *K*_m_ value with **7**, which has a lactone ring, hydroxyl groups on C-5 and C-14, and an aldehyde on C-19. The formation of glucosides of **5** and **7** were confirmed *in planta* by *Agrobacterium*-mediated expression in *N. benthamiana* and LC-MS (Figs. 6; S7). In *E. cheiranthoides*, it has been shown that **5** is a less common cardenolide genin relative to **7** (12). Low levels of **5** *in vivo* may explain why UGT73C44 has such a low *K*_m_ value with this substrate relative to **7**, as it would be necessary to ensure proper substrate specificity under physiological conditions (Fig. 2*C*). Because the pathway intermediate between **5** and **7**, cannogenol (**6**), is commercially unavailable, we do not know how active UGT73C44 or UGT73C45 would be with this substrate. The trend of increasing activity and decreasing *K*_m_ values from **3** to **4** to **5** may continue to **6**, or alternatively, activity with **6** may decline and *K*_m_ may increase, given that **7** exhibited the lowest activity and highest *K*_m_ value of the cardenolide genins. UGT73C44 has higher activity with **3**, which does not have a lactone ring, than with **7**, suggesting that this may contribute to substrate recognition but plays less of a role than hydroxylation of the steroid core. Furthermore, these *in vitro* experiments used N-terminal His-tagged protein, which may affect protein folding, stability, or activity.

While another UGT has been identified that uses **5** as a substrate in the cardenolide-producing plant *Asclepias curassavica* (tropical milkweed), this enzyme, UGT74AN1, has broad substrate specificity and used 53 diverse substrates to form *O*-, *N*-, and *S*-glycosides (37). UGT74AN1 has a low *K*_m_ value of 8.2 μM with **5**, which is similar to the *K*_m_ value of 7.0 μM that we report here for the wallflower UGT73C44 (Fig. 2*C*). Active orthologs of UGT74AN1 have also been identified in *Catharanthus roseus* (Madagascar periwinkle) and *Calotropis gigantea* (giant milkweed) that share 59% and 80% sequence identity with the *A. curassavica* UGT74AN1, but the homologs with the highest similarity in *E. cheiranthoides* share less than 44% identity with the *A. curassavica* gene (37, 38). The UGTs identified here are phylogenetically unrelated to UGT74AN1 (Fig. S2). Therefore, it is unclear whether a UGT74AN1 ortholog is present in wallflower.

As the active sites of UGT73C44 and UGT73C45 vary considerably, despite the fact that they both are active with cardenolides, it is difficult to predict which active site residues confer specificity with steroids (Fig. 7). Having two additional aromatic residues in the UGT73C45 active site may contribute to its activity with a number of flavonoids, and having fewer aromatic residues in the UGT73C44 substrate binding pocket may hinder interactions with flavonoids (Fig. 5). While both active sites have three polar residues, there are two charged residues (Glu199 and Lys422) in UGT73C45 but only uncharged residues in the substrate-binding pocket in UGT73C44. These differences in charge may also contribute to substrate recognition and may allow UGT73C45 to accept monoglycosylated substrates. However, the charged residues, aside from the catalytic Asp, do not interact with the sugar of isoquercetin in our docking results (Fig. 6). Future efforts to empirically solve the structures of these proteins and mutagenesis experiments are needed to confirm the connections among protein structure, substrate recognition, and activity.

Digitoxigenin with glucose as the first sugar has been reported in *E. cheiranthoides* plants, but across the *Erysimum* genus, strophanthidin glycosides typically have a digitoxose or deoxyhexose as the sugar on C-3 (10). This may account for the 19.5-fold higher *K*_m_ value and 16-fold lower activity between **5** and **7** with UGT73C44 when UDP-Glc is the sugar donor (Fig. 2, *B* and *C*). In the *in planta* experiments, we cannot be certain what sugar donor was used since the UDP-sugar profiles likely differ between *N. benthamiana* and *Erysimum* and the LC-MS does not allow us to discriminate between glucose isomers. As UDP-digitoxose is not commercially available, we were unable to test this unique sugar in our experiments. Because UGT73C44 and UGT73C45 have a conserved Gln in the PSPG motif that is found in other UDP-Glc-using UGTs, this supports the use of UDP-Glc as a sugar donor in our assays. However, we cannot rule out the possibility that there are other sugar donors that the enzymes would accept *in vivo*.

UGT73C44 and UGT73C45 are both expressed *in vivo* to high levels in *E. cheiranthoides* and show the highest expression in this species relative to the other *Erysimum* species surveyed, which indicates that other UGTs may contribute to cardenolide glucoside formation (Fig. 3). Follow-up investigations are needed to identify cardenolide pathway UGTs across the *Erysimum* genus, as well as enzymes that add subsequent sugars onto monoglycosylated cardenolides. While we show that digitoxigenin is glucosylated by UGT73C44, we do not know if digitoxigenin glucoside is a substrate for downstream enzymes that hydroxylate the steroid core, or whether core hydroxylation occurs prior to glycosylation for generating products like strophanthidin glucosides. This may be possible to test once additional enzymes have been identified. Additionally, genetic manipulation of *UGT73C44* and *UGT73C45* in *E. cheiranthoides via* knockdown or knockout would provide further confirmation of the contributions of these UGTs to the cardenolide pathway and to plant metabolism and physiology more broadly, especially for UGT73C45.

In summary, we present a cardenolide pathway-specific UGT from wormseed wallflower that has high activity and specificity for 14-anhydrodigitoxigenin and digitoxigenin (Fig. 2). We also present data for a neighboring UGT, UGT73C45, which has broad substrate specificity and can also generate cardiac glycosides *in vitro.* Because there is interest in generating and testing structurally diverse cardenolide analogs to test as anticancer and antiviral agents and improved cardiotonic drugs, identification of UGT73C44 represents a stepping stone toward the reconstitution of the cardenolide pathway in heterologous systems like yeast or *N. benthamiana* (39, 40, 41, 42, 43). Our findings also contribute to solving the decades-long question of how plants synthesize the medically and ecologically relevant cardenolides.

## Experimental procedures

### Candidate gene identification

Raw RNA-sequencing reads from 48 *Erysimum* species (PRJNA563696) (10) and *E. cheiranthoides* tissues (PRJNA1015726) (14) were downloaded from the NCBI Short Read Archive. Read counts were pseudoaligned to the transcriptome associated with *E. cheiranthoides* genome annotation v2.1 (44) using kallisto (45), and coexpression networking was performed on the species expression data as described in Younkin *et al.* (12). Heatmaps were made in R Statistical Software 4.4.1 (46) with the pheatmap (47) and RcolorBrewer (48) packages.

### Protein expression and purification

*Erche07g009482.1* and *Erche07g009500.1* were codon-optimized for expression in *E. coli*, synthesized, and cloned into the pET28a expression vector by Twist Bioscience. Each pET28a construct was transformed into *E. coli* BL21 (DE3) cells (New England Biolabs) or C43 cells (Sigma Aldrich). For protein expression, one-L cultures in Terrific Broth started with 25 ml of an overnight culture at 37 °C. Once the *A*_600nm_ reached 0.6 to 0.8, the culture temperature was lowered to 16°C and 1 mM IPTG was added. After an approximately 16-h incubation, cells were pelleted by centrifugation (5000*g*; 15 min) and resuspended in 40 ml of lysis buffer (50 mM Tris (pH 8.0), 500 mM NaCl, 20 mM imidazole, 10% glycerol, and 1% Tween-20). Cells were lysed by sonication, and then cell debris was pelleted by centrifugation (13,000*g*; 60 min). The supernatant was passed over a Ni^2+^-nitrilotriacetic acid column equilibrated in the lysis buffer. The resin was then washed (50 mM Tris (pH 8.0), 500 mM NaCl, 20 mM imidazole, and 10% glycerol) and bound His-tagged protein was eluted (50 mM Tris (pH 8.0), 500 mM NaCl, 250 mM imidazole, and 10% glycerol). Protein concentration was determined using Bradford solution (Bio-Rad) with bovine serum albumin as the standard, and protein aliquots were stored at −80 °C. Protein purity was visually inspected by SDS-PAGE (Fig. S12).

### Luminescence kinetics assay

UGTs were assayed using the UDP-Glo glycosyltransferase assay (Promega). As UDP is released from UDP-Glc enzymatically, a UDP Detection Reagent converts UDP to ATP and luciferase/luciferin then generates light. A reaction buffer was prepared so that the final concentrations in each well were 50 mM Tris (pH 7.5) and 5 mM MgCl_2_. Tested substrates included UDP-Glc, epipregnanolone, 14-anhydrodigitoxigenin, digitoxigenin, and strophanthidin, which were purchased from Sigma Aldrich or Cayman Chemical. Product formation was calculated in terms of nmol of product per milligram of enzyme using a UDP standard curve ranging from 0 to 25 μM with four concentrations in between (n = 3) (Fig. S13). For initial comparisons, 0.1 mM of substrates and 1 mM UDP-Glc were incubated with 350 ng of protein for 10 min at 37°C. Protein concentration was adjusted to 350 ng for determining Michaelis-Menten parameters for UDP-Glc (0–2 mM), digitoxigenin (0–0.1 mM), 14-anhydrodigitoxigenin (0–0.1 mM), and 1250 ng with epipregnanolone (0–75 μM) and strophanthidin (0–1 mM) with at least six concentrations. Each enzyme was assayed in triplicate with 1 mM UDP-Glc or 0.4 mM digitoxigenin for assays where UDP-Glc was varied. Product formation was also assessed with 0.4 mM digitoxigenin and UDP-glucuronic acid (Fig. S14). The 25 μl reactions were initiated by the addition of substrates, and the reactions were incubated at 37 °C for 10 min for UDP-Glc, digitoxigenin, and 14-anhydrodigitoxigenin and 12 min for epipregnanolone and strophanthidin. Reactions were quenched by the addition of 25 μl of UDP Detection Reagent according to the manufacturer's technical manual. Luminescence readings were measured using a BioTek Cytation one plate reader.

### Product detection by LC-MS

A reaction buffer was prepared so that the final concentrations in each 250 μl reaction were 50 mM Tris (pH 7.5) and 5 mM MgCl_2_. Substrates (100 μM epipregnanolone, 1 mM 14-anhydrodigitoxigenin, 1 mM digitoxigenin, 1 mM strophanthidin, 0.5 mM quercetin, 0.5 mM isoquercetin, 0.5 mM quercimeritrin, 1 mM hesperidin, 0.5 mM 3β,5α-THDOC, or 1 mM ouabain) and 1 mM UDP-Glc and were incubated with 5 μg of protein for 15 min at 37 °C. After, the reactions were quenched with 1 μl of formic acid, and 750 μl of 100% methanol was added. Reactions were vortexed, incubated for a minimum of 1 h at −20 °C to precipitate the protein, and then centrifuged at max speed for 5 min before being loaded into LC-MS vials. The samples were analyzed using an Agilent 6230B TOF LC/MS. Chromatographic separation was performed with an Agilent Zorbax Eclipse Plus C18 column (2.1 × 50 mm, 1.8 μm particle size) at 40 °C. A 1 μl injection volume was used. The mobile phases consisted of water with 0.1% formic acid (A) and acetonitrile (B). The flow rate was set to 0.5 ml/min with the following gradient: 95% A decreasing to 5% A over 10 min, held at 5% A for 2 min, then returned to 95% A over 1 min, followed by a 2-min re-equilibration at 95% A, for a total run time of 15 min. The HPLC system included an Agilent G1312 B binary pump, G7129 A autosampler, G7130 A column oven, and G1315 C diode array detector. The TOF mass spectrometer operated in positive ion mode with a reference-corrected dual electrospray Agilent Jet Stream source. Source parameters were as follows: gas temperature, 300 °C; gas flow, 11 L/min; nebulizer pressure, 55 psig; sheath gas flow, 12 L/min at 400 °C; and nozzle voltage, 1000 V. The capillary voltage was set to 4000 V, with a fragmentor voltage of 175 V, skimmer voltage of 65 V, and octopole RF peak at 750 V. Spectra were acquired in the m/z range of 75 to 1100 at a rate of 1.1 spectra/s. Data were analyzed using Agilent MassHunter Quantitative Analysis software. All substrates were included as standards as were expected products when they were commercially available (Fig. S4).

### Expression of UGT73C44 in *N. benthamiana*

*N. benthamiana* plants were grown on ProMixBX soil in a Conviron Gen1000 growth chamber on a 16-h day length with 50% humidity. Daytime temperature was set to 25 °C, and nighttime temperature was set to 23 °C. The *Erche07g009482* gene that encodes UGT73C44 was cloned from *Erysimum* cDNA into the pEAQ-HT-DEST1 plasmid and transformed into *Agrobacterium tumefaciens* GV3101 using the freeze-thaw method (49, 50). The gene that encodes GFP was cloned into pEAQ-HT-DEST2 to use as a positive control. Colonies were used to start 5 ml LB cultures supplemented with kanamycin (50 μg/ml) and gentamycin (30 μg/ml), and the cultures were incubated at 28°C and 250 rpm. The following day, 1 ml of these cultures was used to inoculate 50 ml LB cultures with kanamycin and gentamycin. On the day of the infiltrations, five-week-old *N. benthamiana* plants were placed in a dark space for 2 hours prior to infiltrations, and 50 ml cultures were centrifuged at 3000*g* for 30 min and cell pellets were resuspended in MMA buffer (10 mM MES (pH 5.6), 10 mM magnesium chloride, 100 μM acetosyringone). Resuspended cells were diluted to an OD_600_ of 0.42 to 0.45 and incubated in MMA buffer at room temperature for 1 hour prior to infiltrations. The underside of leaves was infiltrated with the *Agrobacterium* expressing either *UGT73C44* or *GFP* using a needless syringe. Four days later, these same leaves were infiltrated with 100 μM substrates (digitoxigenin, strophanthidin, or epipregnanolone) in 0.1% DMSO or a solvent-only control. On Day 5, five leaves were hole-punched for each gene–substrate combination to collect approximately equal masses (roughly 75 mg) of plant tissue; only four leaves were collected for the gene-epipregnanolone combinations. Leaf samples were flash frozen in liquid nitrogen and pulverized using steel beads in a Bead Genie homogenizer. Afterward, samples were incubated in 75% MeOH for 10 min at room temperature, centrifuged at 10,000*g* for 5 min, and transferred to LC-MS vials. Products were detected using the same LC-MS methods and parameters as mentioned above.

### Protein visualization and molecular docking

Structural models of all proteins were generated using ColabFold v1.5.5 (51) (Fig. S15), and AutoDock Vina (ver. 1.1.2) was used to add digitoxigenin, strophanthidin, and epipregnanolone to the active site of UGT73C44 and digitoxigenin, quercetin, and isoquercetin to the active site of UGT73C45 (grid box dimensions of 40 x 40 x 40 Å; exhaustiveness set to 8) (52, 53). PyMOL (ver. 2.5.7) was used to visualize the results (https://www.pymol.org/2/). UDP-Glucose was added to the dimers using AlphaFill (54).

### Phylogenetic analysis

Homologous sequences were selected based on previous phylogenetic analyses of *Arabidopsis* UGT73C family enzymes, and the UGT74AN from *A. curassavica* was used as an out-group (37, 55). Selected *E. cheiranthoides* sequences (genome v2.0) had greater than 50% sequence similarity to the *Arabidopsis* orthologs (erysimum.org). Amino acid sequences were aligned using MAFFT version 7, and phylogenies were inferred using the IQ-TREE web server with default parameters and 10,000 ultrafast bootstrap replicates (56, 57, 58, 59). The best fit model according to Bayesian Information Criterion was the JTT model with a discrete Gamma model with four rate categories (60, 61). The maximum likelihood dendrogram was visualized using FigTree (version 1.4.4).

## Data availability

All data are contained within the manuscript.

## Supporting information

This article contains supporting information.

## Conflict of interest

The authors declare that they have no conflicts of interest with the contents of this article.
