# Supplementary material for: Identification of UDP-dependent glycosyltransferases in the wallflower cardenolide biosynthesis pathway
Source: J Biol Chem. 2025 Apr 30;301(6):108565. doi: 10.1016/j.jbc.2025.108565 (PMC12155548; doi:10.1016/j.jbc.2025.108565)
Supplement: Supporting information [file mmc1.pdf]

SUPPORTING INFORMATION

Identification of UDP-dependent glycosyltransferases in the wallflower cardenolide biosynthesis pathway

Owen S. Patrick[a], Gordon C. Younkin [b,c], Rebecca G. Brody[a], Jessica W. Hem[a], Georg Jander [c], Cynthia K. Holland[a*]

[a]Department of Biology, Williams College, Williamstown, MA 01267 USA

[b]Plant Biology Section, School of Integrative Plant Science, Cornell University, Ithaca, NY 14853 USA

[c]Boyce Thompson Institute, Ithaca, NY 14853 USA

[*]**Corresponding author:** ckh2@williams.edu

Includes Supporting Figure S1-15

Figure S1. Amino acid sequences of UGTs. Sequences were determined using the *Erysimum cheiranthoides* genome and transcriptome from erysimum.org.

>Erche07g009482.1
MASETNHQFLPPLHFVLFPFMAQGHMIPMVDISRILAQHGVTITIVTTPQNAGRFENVLSRAIQS
GLPINIVQIKFPSNESGSSEGRENLDSLDSLDLDTTFSFFKASSVWEESVEKLLKEIQPRPNCIIA
DMCLPYTSRVAKNLGIPKIVFHAMGCFDILCMHVSFQNLEFLETIESEHEYFVIPNFPGRTKFTKT
QIPMILATGEWKEFVDAMVEADNSSYGVIINTFEELETAYIRDYKKARADKVWSIGPVSLCNKVG
EDKFERGKKAAIDQDECLKWLDSREECSVLYVCLGSICNLPLSQLKEIGLGLEESQRPFIWVIRG
WEKNNELVVWISESGFKERIKERGLLIMGWAPQMLILSHPAIGGFFTHCGWNSTLEGITSGVPL
LTWPLFGDQFCNDKLAVQILKVGVAAGVEDLMQWGEEEKIGVLVNKEGVKKAVEELMGDSND
AKEIRKRVKELGELAHKAVEEGGSSRSNIMAFLQDITQLAQSKND-

>Erche07g009500.1
MVSKTTNESYPLHFVLFPFMAQGHMIPMIDIARLFAQRGVMVTIVTTPHNAGRFKNVLNRAIES
GLPINLVQVKFPYQEAGLQEGQENVDCLETMEKMASFFKVIKLLEEPAQKLIEEMSPRPSCLISD
FCLPYTSKIAKKFNIPKILFHGMSCFCLLCMHVLRKNLEILENLKSDKEHFIVPYFPDRVEFTRPQ
VPVETYVPSDFKEFLDDLVEAEKTSYGVIVNTFEELEPAYAKDFKEVRSGKAWTIGPVSLCNKA
GADKAERGNKSDIDQDECLKWLDSKEPGSVLYVCLGSICNLPLPQLKELGLGLEESQRPFIWVI
RGWEKYKELVEWFSESGFEERVKDRGLLIKGWSPQMIILSHHSVGGFLTHCGWNSTLEGITAG
LPLLTWPLFADQFCNEKLVVQVLKAGVRVGVEQPMKWGEEDKIGVLVDKEGVKKAVEELMGE
SDDAKERRKRAKELGGLAHKAVEEGGSSHSNITFLLQDIMQLAQSKN-

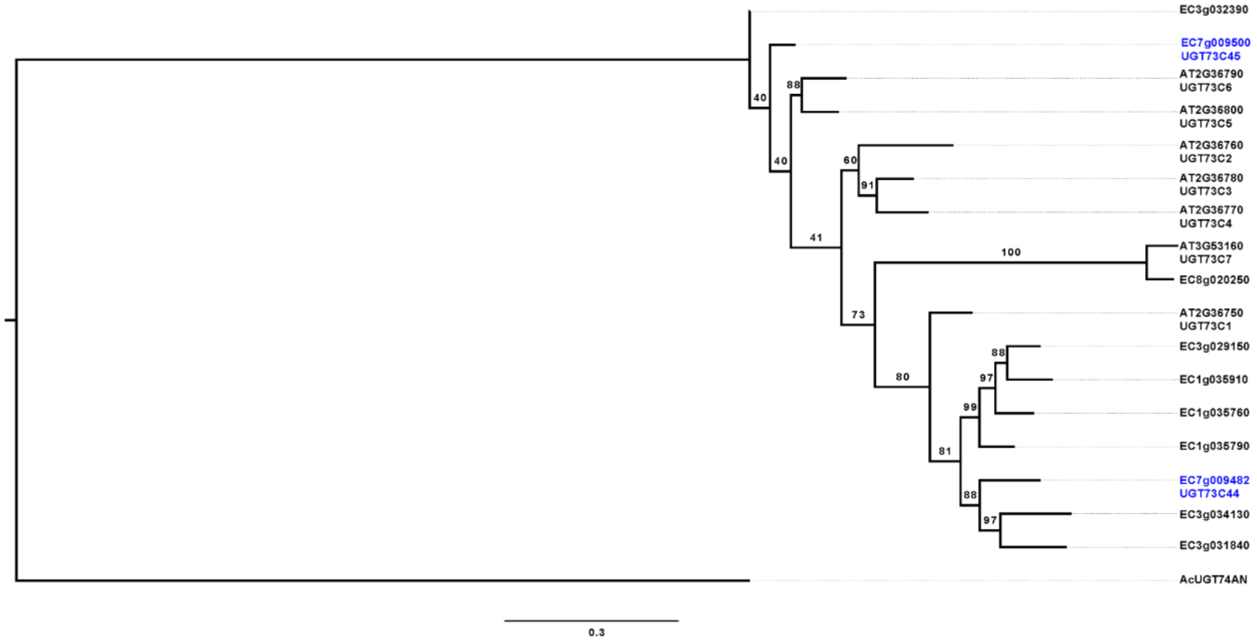

Figure S2. Phylogenetic analysis of UGTs. The dendrogram of *Arabidopsis thaliana* (AT) and *Erysimum cheiranthoides* (EC) UGT73C homologs shows an evolutionary relationship among the enzymes characterized here (blue) and across the two Brassicas. The previously characterized UGT74AN from *Asclepias curassavica* (AN) was included as an outgroup.

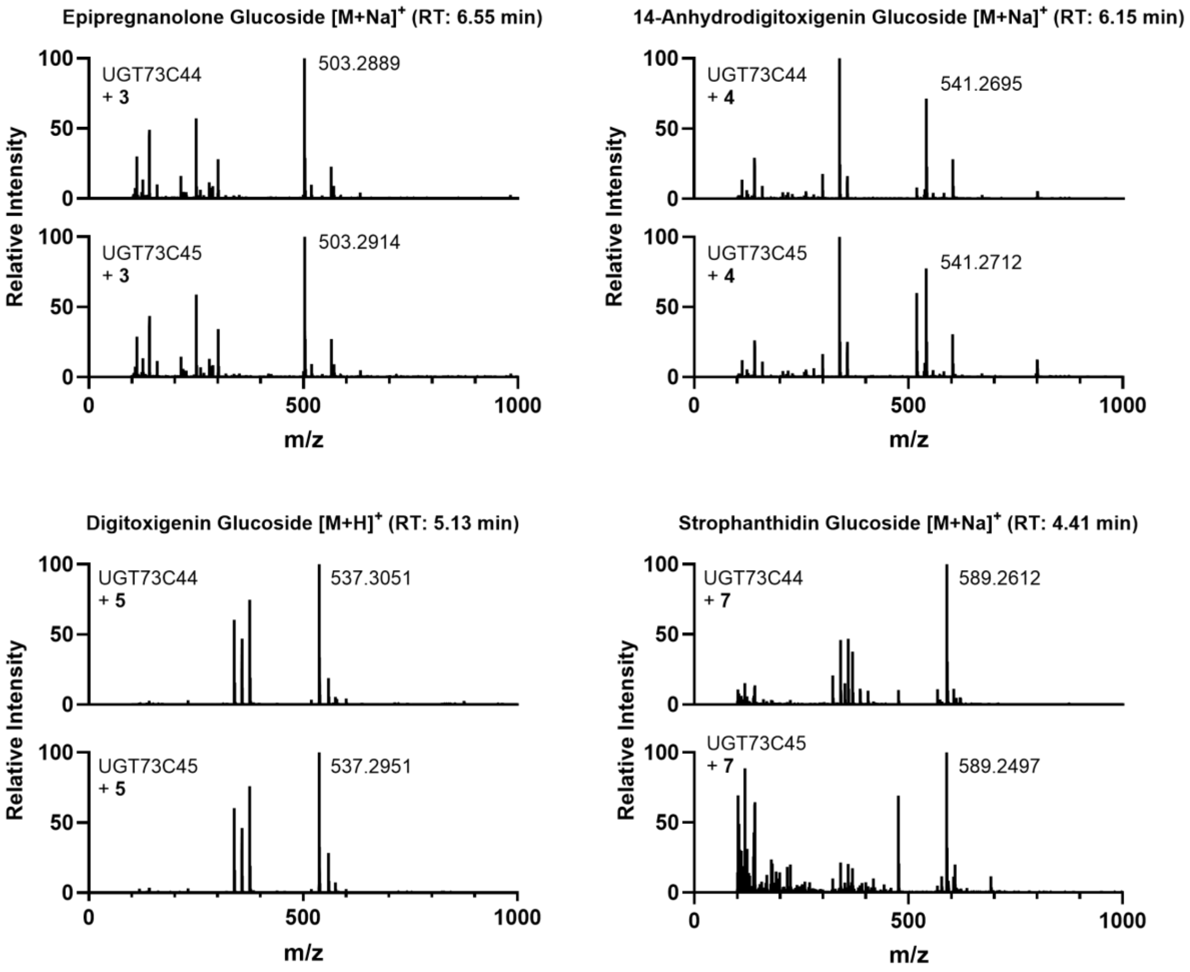

Figure S3. Mass spectra for glucosylated cardenolide pathway intermediates formed by UGT73C44 and UGT73C45 *in vitro*. Spectra represent the enzymatic products formed following incubation with **3** (epipregnanolone), **4** (14-anhydrodigitoxigenin), **5** (digitoxigenin), or **7** (strophanthidin). Data shown are representative of three replicates.

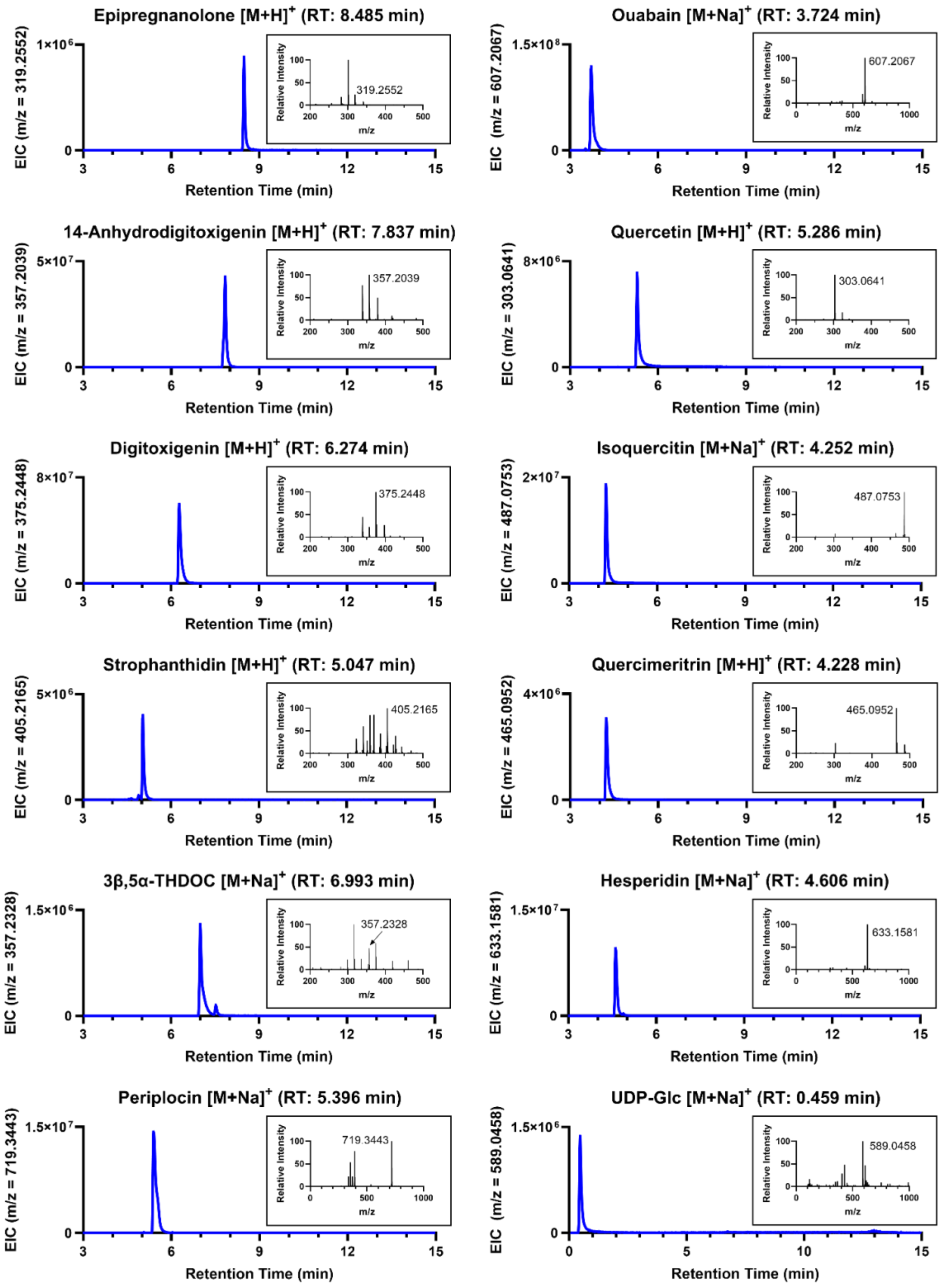

Figure S4. Extracted ion chromatograms and spectra of all substrate standards. All standards were dissolved in 75% MeOH to a concentration of 500 µM, with the exception of epipregnanolone, which was dissolved to 100 µM due to solubility limitations.

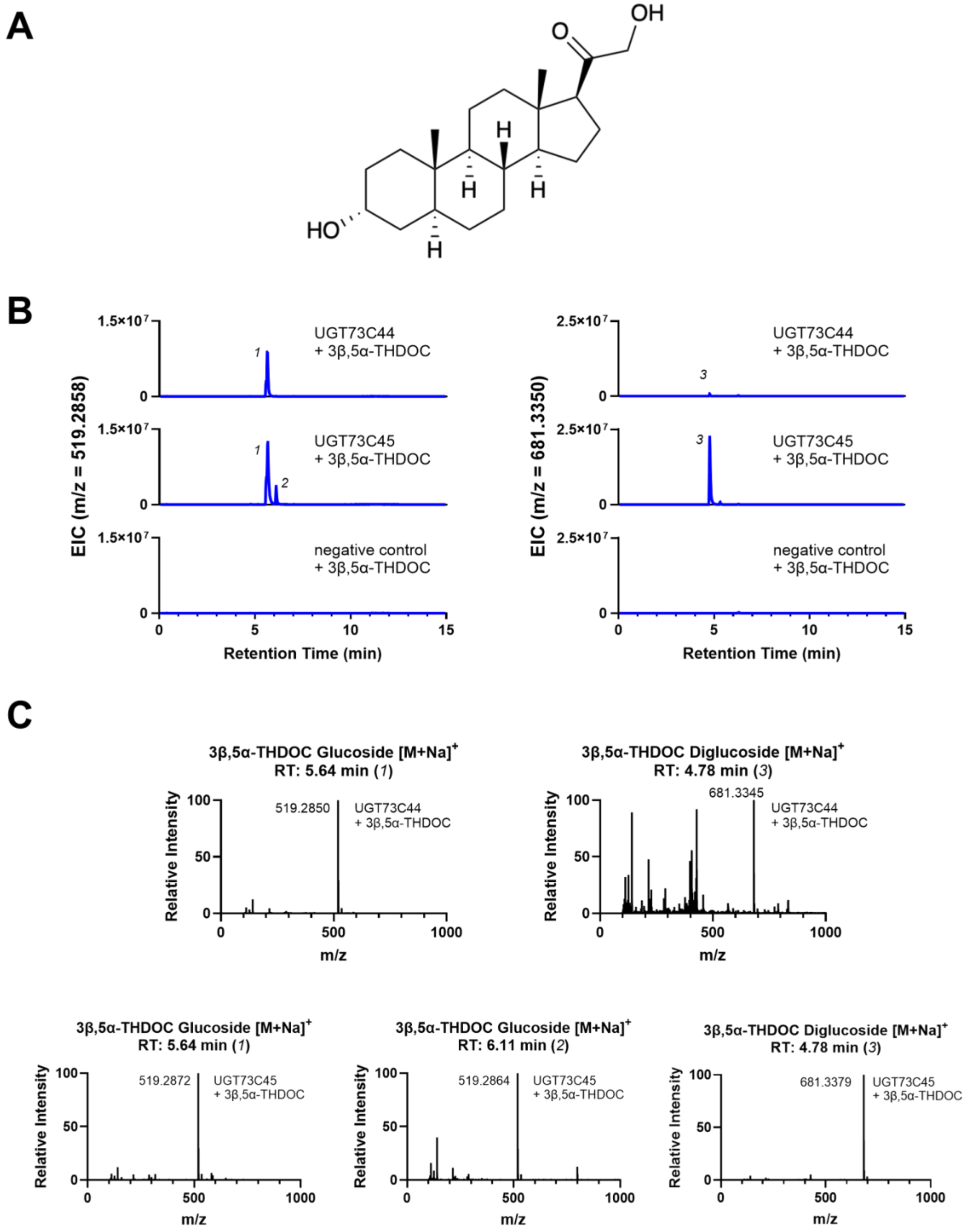

Figure S5. Detection of glucosylated and diglucosylated 3β,5α-THDOC produced by UGT73C44 and UGT73C45 *in vitro*. *A*, Structure of 3β,5α-THDOC. *B*, Extracted ion chromatograms (EICs) at m/z values corresponding to $[M+Na]^+$ adducts of glucosylated and diglucosylated 3β,5α-THDOC. *C*, Mass spectra of the major peaks observed in the EICs. Data shown are representative of three independent replicates.

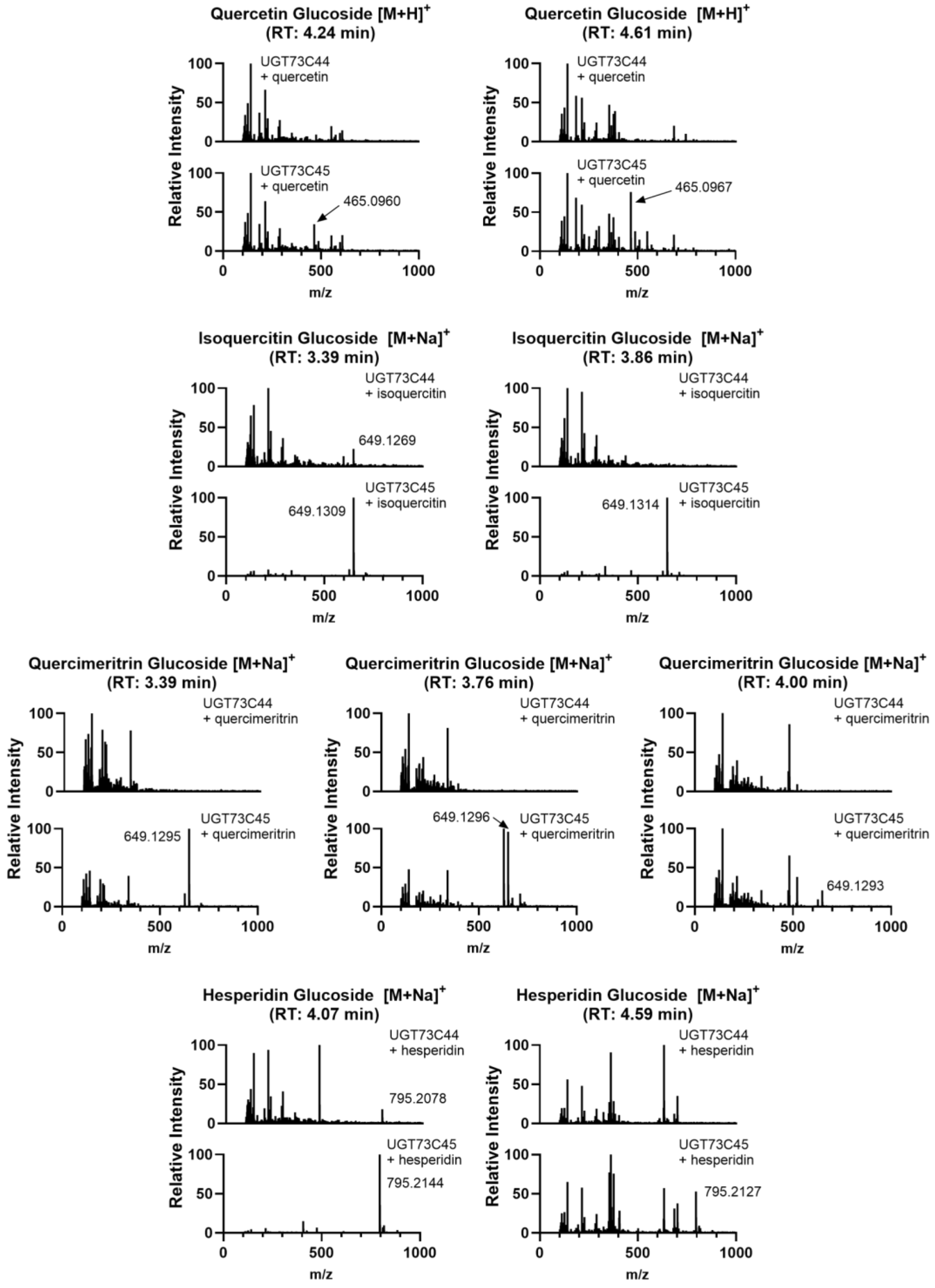

Figure S6. Mass spectra for glucosylated flavonoids formed by UGT73C44 and UGT73C45 *in vitro*. Spectra correspond to the chromatograms presented in Figure 5 and are representative of three replicates.

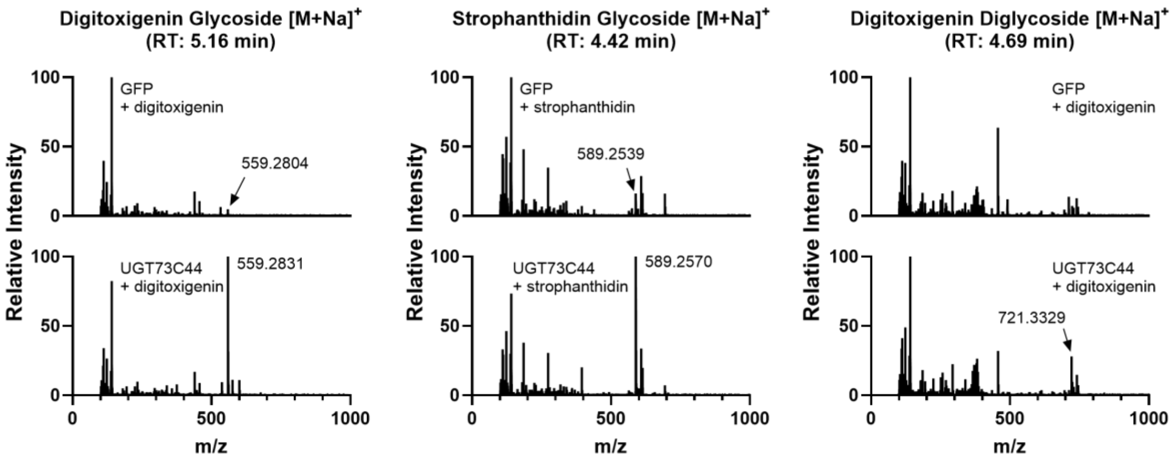

Figure S7. Mass spectra for glycosylated and diglycosylated cardenolides produced by UGT73C44 and UGT73C45 *in planta*. Spectra correspond to the chromatograms presented in Figure 6 and are representative of five replicates.

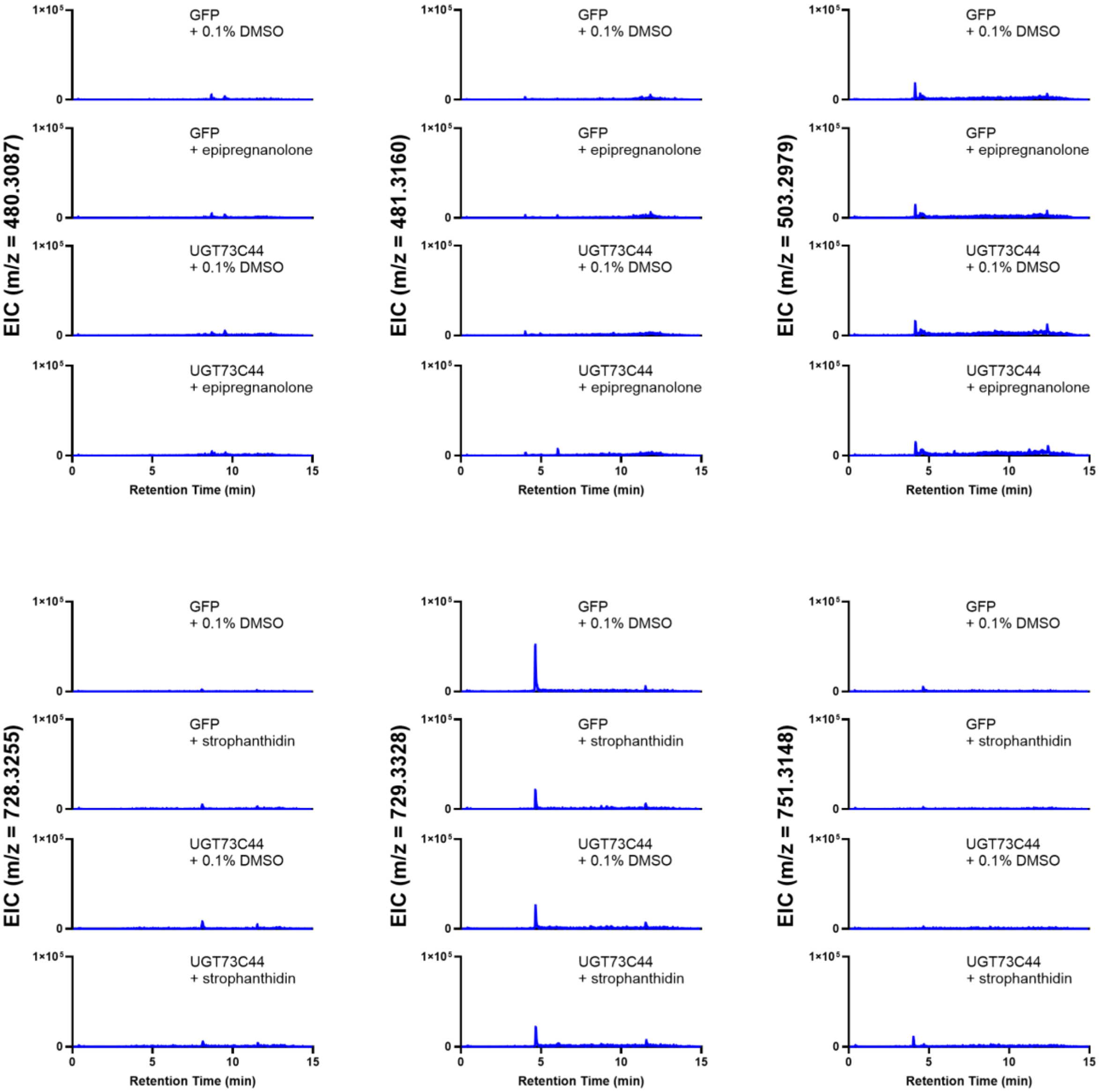

Figure S8. Extracted ion chromatograms (EICs) at predicted m/z for glycosylated epipregnanolone and diglycosylated strophanthidin when incubated with UGT73C44 *in planta* via expression in *N. benthamiana*. GFP expression serves as a control for gene expression, and 0.1% DMSO serves as a solvent infiltration control. EICs are included for predicted m/z values that correspond to the M^+ peak, as well as the $[M+H]^+$ and $[M+Na]^+$ adducts of epipregnanolone glucoside and strophanthidin diglucoside. Data are representative of four replicates for epipregnanolone and five replicates for strophanthidin.

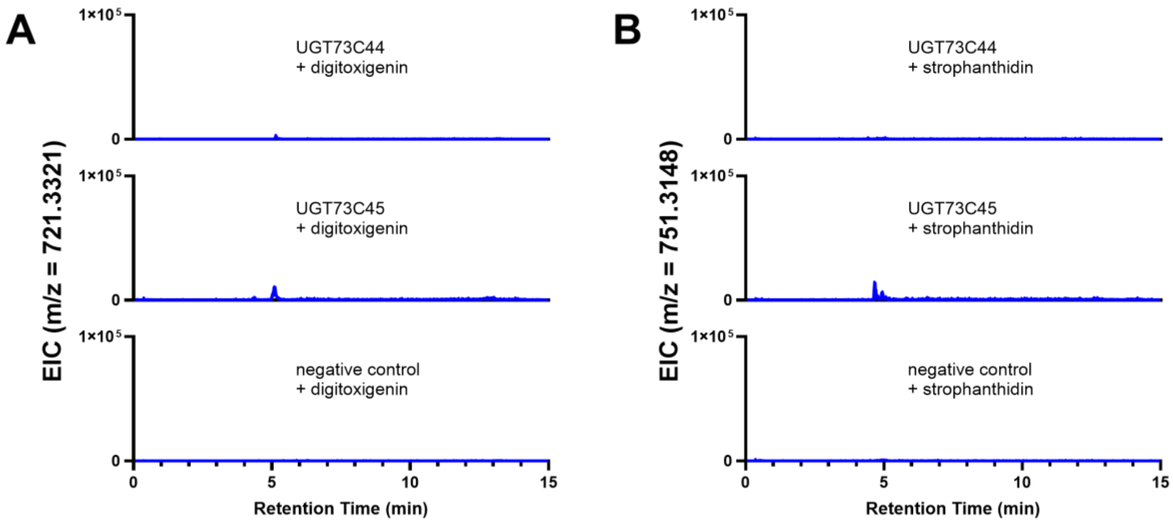

Figure S9. Detection of diglucosylated digitoxigenin and strophanthidin produced by UGT73C44 and UGT73C45 *in vitro*. *A*, Extracted ion chromatogram (EIC) for an m/z that corresponds to diglucosylated digitoxigenin $[M+Na]^+$ detected at a retention time of 5.10 min. *B*, EIC for an m/z that corresponds to diglucosylated strophanthidin $[M+Na]^+$ detected at a retention time of 4.67 min. Data are representative of three replicates.

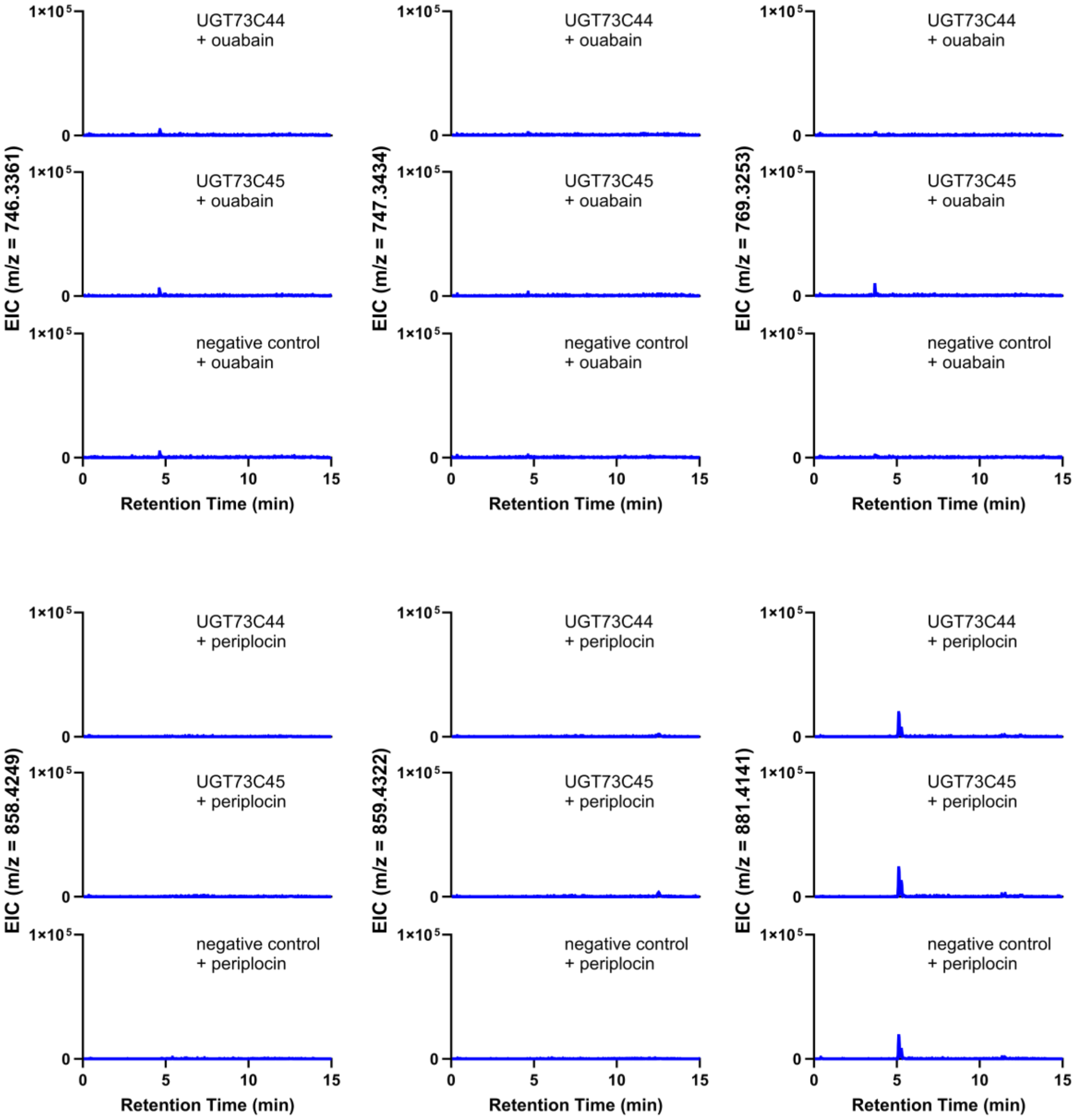

Figure S10. Extracted ion chromatograms (EICs) at predicted m/z for glucosylated ouabain and periplocin formed by UGT73C44 and UGT73C45 *in vitro*. EICs for predicted m/z values that correspond to the M^+ peak and $[M+H]^+$ and $[M+Na]^+$ adducts of ouabain glucoside and periplocin glucoside are included. No corresponding product ions were detected under the experimental conditions.

```
AcUGT74AN       -MGTIEISSPRKTHILAFPFPAKGHINPMLHLCNRLASKGFRVSFITTISTYKDAKNKIE    59
UGT73C44        MASETNHQFLPPLHFVLFPFMAQGHMIPMVDISRILAQHGVTITIVTTPQNAGRFENVLS    60
UGT73C45        MVSKT-TNESYPLHFVLFPFMAQGHMIPMIDIARLFAQRGVMVTIVTTPHNAGRFKNVLN    59
                  .    .     *:: *** *:**: **:.:.. :*.:*. ::::**   .     :* :.

AcUGT74AN       S--KSGGLINLESI---------PDGTEKK-----LSMSHYFNKFR--DSVTENVSGIIE    101
UGT73C44        RAIQSGLPINIVQIKFPSNESGSSEGRENLDSLDSLDLDTTFSFFKASSVWEESVEKL--    118
UGT73C45        RAIESGLPINLVQVKFPYQEAGLQEGQENVDCLETME--KMASFFKVIKLLEEPAQKL--    115
                   :**  **: .:              :* *:       :.     . *:  .    * .. :

AcUGT74AN       KYKLGVDFPPPKVFIYDSTMPWMLDVAHGHGILGASLFTQPCCVSAVYYHMLQGTLDS--    159
UGT73C44        ---LKEIQPRPNCIIADMCLPYTSRVAKNLGIPKIV-FHAMGCFDILCMHVSFQNLEFLE    174
UGT73C45        ---IEEMSPRPSCLISDFCLPYTSKIAKKFNIPKIL-FHGMSCFCLLCMHVLRKNLEILE    171
                   :    * *. :* *  :*:   :*:  .*      *    *.  :  *:    .*:

AcUGT74AN       -SSSSSRVSLLPCLPPLE---DRDLPEFDYFKE-DGEFVSNLLTNQFLNIDKID-YVLFN    213
UGT73C44        TIESEHEYFVIPNFPGRTKFTKTQIPMI-LATGEWKEFVDAM-----VEADNSSYGVIIN    228
UGT73C45        NLKSDKEHFIVPYFPDRVEFTRPQVPVETYVPSDFKEFLDDL-----VEAEKTSYGVIVN    226
                  .*. .  ::* :*        ::*            **:. :      :: :: .  *:.*

AcUGT74AN       TFEKLEAEIANWMS--SKWKILTIGPTAPTPVGAALETEEERINNYVLETNTEVCMKWLN    271
UGT73C44        TFEELETAYIRDYKKARADKVWSIGPVSLCNKVGE--DKFER--GKKAAIDQDECLKWLD    284
UGT73C45        TFEELEPAYAKDFKEVRSGKAWTIGPVSLCNKAGA--DKAER--GNKSDIDQDECLKWLD    282
                ***:**     .  .     *  :***.:      .    : **  .      : : *:***:

AcUGT74AN       EREPNSVIYVSFGSIASLTELQMEEILEALLAANFNFLWVVREEERAKLPNYSESSSGII    331
UGT73C44        SREECSVLYVCLGSICNLPLSQLKEIGLGLEESQRPFIWVIRGWEKNNELVVWISESGFK    344
UGT73C45        SKEPGSVLYVCLGSICNLPLPQLKELGLGLEESQRPFIWVIRGWEKYKELVEWFSESGFE    342
                .:*   **:**.:***..*    *::*:   .*   ::   *:**:*   *: :       *.**:

AcUGT74AN       T-VTGKLGLIVNWCPQLEVLSHESLACFMTHCGWNSTLEAISSGVVMIGVPQWVDQTTNA    390
UGT73C44        ERIKERGLLIMGWAPQMLILSHPAIGGFFTHCGWNSTLEGITSGVPLLTWPLFGDQFCND    404
UGT73C45        ERVKDRGLLIKGWSPQMIILSHHSVGGFLTHCGWNSTLEGITAGLPLLTWPLFADQFCNE    402
                  :. :   **  .*.**: :*** ::.  *:**********.*::*: ::   * : **   *

AcUGT74AN       KFIEDVWKIGVRVKNNS------NGENGGLVKKEEIERCIKEV-CESEKGKELKRNAMKW    443
UGT73C44        KLAVQILKVGVAAGVEDLMQWGEEEKIGVLVNKEGVKKAVEELMGDSNDAKEIRKRVKEL    464
UGT73C45        KLVVQVLKAGVRVGVEQPMKWGEEDKIGVLVDKEGVKKAVEELMGESDDAKERRKRAKEL    462
                *:   :: * **  .   :.       : : * **.** :::.::*:   :*:..** ::.. :

AcUGT74AN       KELATEAVSEGGSSDTNLDYFASTLLFY------   471
UGT73C44        GELAHKAVEEGGSSRSNIMAFLQDITQLAQSKND   498
UGT73C45        GGLAHKAVEEGGSSHSNITFLLQDIMQLAQSKN-   495
                  ** :**.***** :*:   : . :
```

PSPG motif
Catalytic dyad
UGT73C44 active site residue
UGT73C45 active site residue

Figure S11. Sequence alignments of UGTs. EcUGT73C44 and EcUGT73C45 sequences were aligned along with UGT74AN from *Asclepias curassavica* using Clustal Omega v.1.2.4.

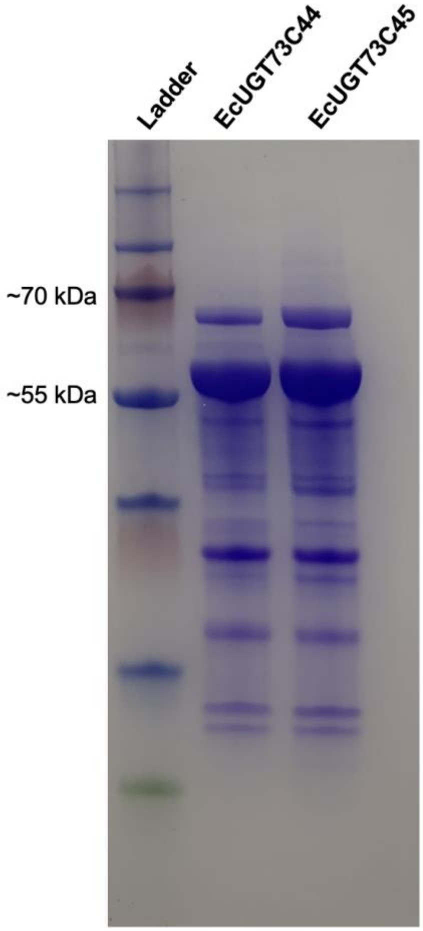

Figure S12. SDS-PAGE gel of Ni-affinity purified EcUGT73C44 and EcUGT73C45. Both UGT73C44 and UGT73C45 are expected to be 58 kDa. The PageRuler Plus Prestained Protein Ladder (ThermoScientific) was used as a size standard. The gel was stained with Coomassie Blue and after destaining, the gel was imaged using a white background.

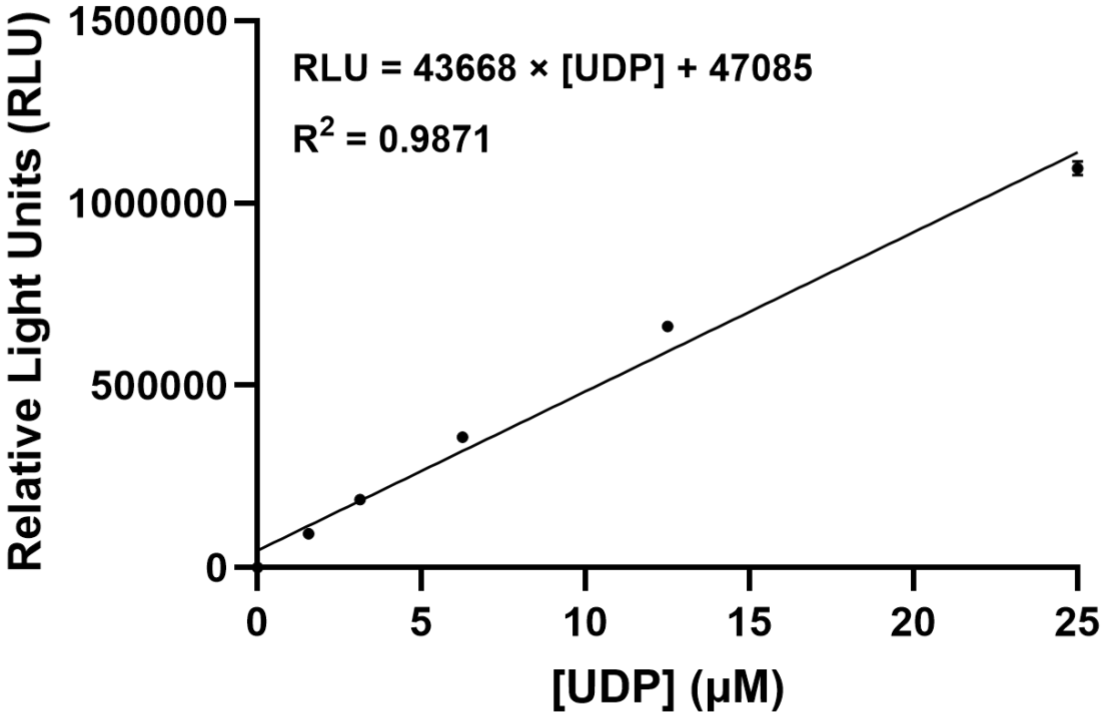

Figure S13. Representative UDP standard curve for UDP-Glo™ Glycosyltransferase assay. Luminescence values were collected for six concentrations of UDP ranging from 0 to 25 μM. Error bars are too small to visualize for most points and represent ± 1 standard deviation (n = 3).

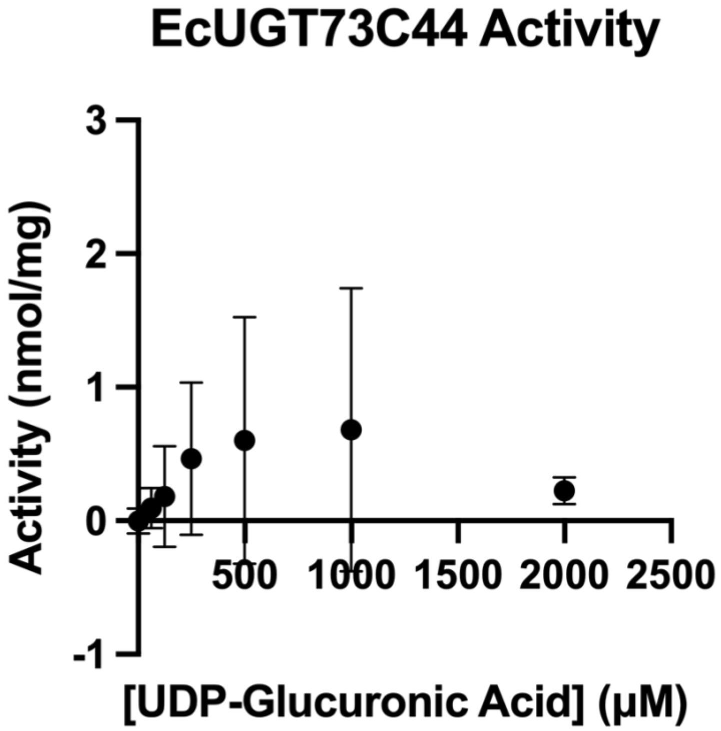

Figure S14. EcUGT73C44 activity UDP-Glucuronic Acid as a sugar donor. Activity of UGT73C44 with 0-2 mM UDP-Glucuronic Acid was determined using 350 ng of enzyme and 0.4 mM digitoxigenin incubated for 10 min at 37°C. Error bars represent standard deviation, and n=3 for all concentrations.

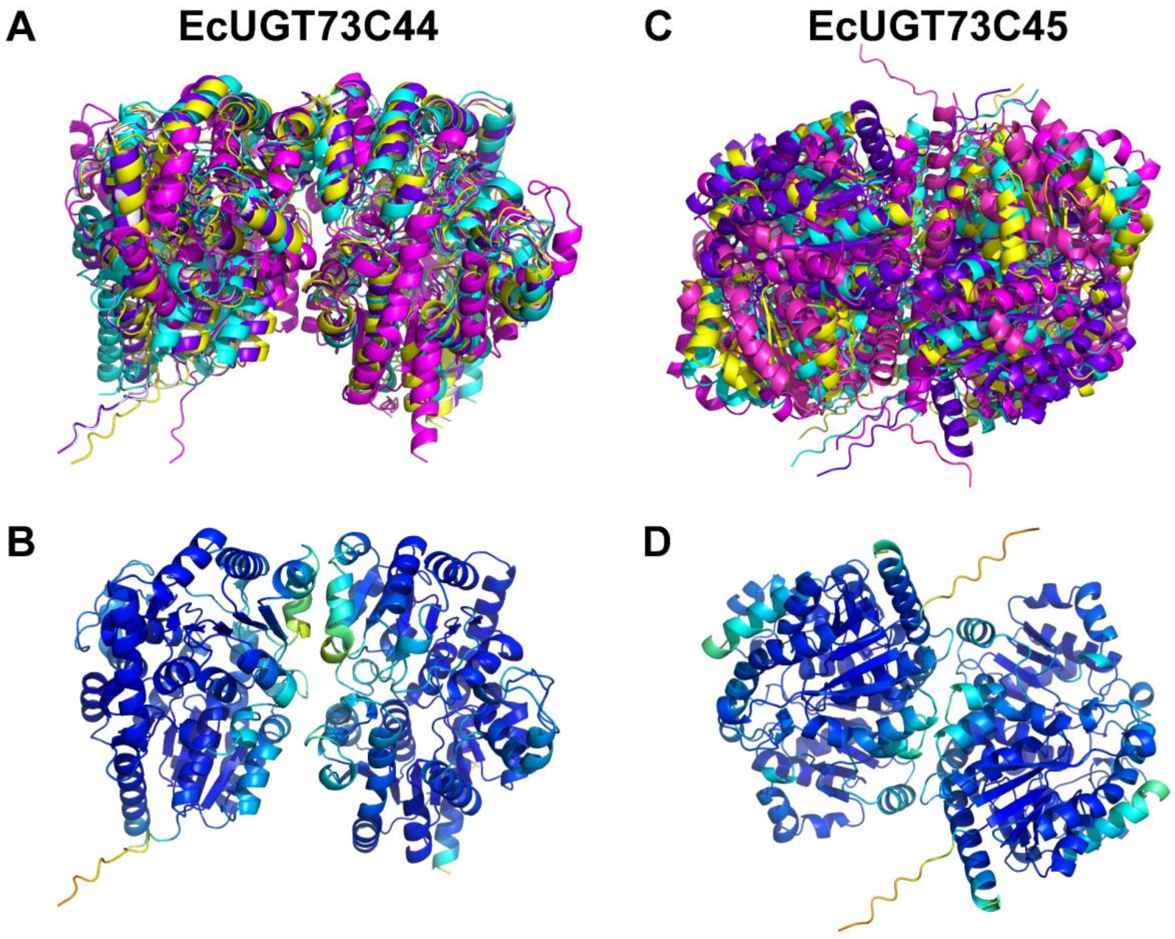

Figure S15. Structural models of EcUGT73C44 and EcUGT73C45. *A*, An overlay of the five best AlphaFold models of EcUGT73C44, as well as *B*, the single best AlphaFold structure shown in colors of pLDDT values. Values colored dark blue have a very high pLDDT of >90; light blue represents a pLDDT value between 90 and 70; yellow represents a pLDDT value between 70 and 50; and orange represents a pLDDT value less than 50. *C*, An overlay of the five best AlphaFold models of EcUGT73C45, as well as *D*, the single best AlphaFold structure shown in colors of pLDDT values.
